# Testing the relationship between microbiome composition and flux of carbon and nutrients in Caribbean coral reef sponges

**DOI:** 10.1186/s40168-019-0739-x

**Published:** 2019-08-29

**Authors:** Shelby E. Gantt, Steven E. McMurray, Amber D. Stubler, Christopher M. Finelli, Joseph R. Pawlik, Patrick M. Erwin

**Affiliations:** 1 Center for Marine Science and Department of Biology and Marine Biology, University of North Carolina Wilmington, Wilmington, NC 28409 USA; 2 Biology Department, Occidental College, 1600 Campus Road, Los Angeles, CA 90041 USA

**Keywords:** Holobiont, Microbiomes, HMA-LMA, POC, DOC, Ammonium (NH_4_), Benthic-pelagic coupling, Sponge-loop

## Abstract

**Background:**

Sponges are important suspension-feeding members of reef communities, with the collective capacity to overturn the entire water column on shallow Caribbean reefs every day. The sponge-loop hypothesis suggests that sponges take up dissolved organic carbon (DOC) and, via assimilation and shedding of cells, return carbon to the reef ecosystem as particulate organic carbon (POC). Sponges host complex microbial communities within their tissues that may play a role in carbon and nutrient cycling within the sponge holobiont. To investigate this relationship, we paired microbial community characterization (16S rRNA analysis, Illumina Mi-Seq platform) with carbon (DOC, POC) and nutrient (PO_4_, NO_x_, NH_4_) flux data (specific filtration rate) for 10 common Caribbean sponge species at two distant sites (Florida Keys vs. Belize, ~ 1203 km apart).

**Results:**

Distance-based linear modeling revealed weak relationships overall between symbiont structure and carbon and nutrient flux, suggesting that the observed differences in POC, DOC, PO_4_, and NO_x_ flux among sponges are not caused by variations in the composition of symbiont communities. In contrast, significant correlations between symbiont structure and NH_4_ flux occurred consistently across the dataset. Further, several individual symbiont taxa (OTUs) exhibited relative abundances that correlated with NH_4_ flux, including one OTU affiliated with the ammonia-oxidizing genus *Cenarchaeum*.

**Conclusions:**

Combined, these results indicate that microbiome structure is uncoupled from sponge carbon cycling and does not explain variation in DOC uptake among Caribbean coral reef sponges. Accordingly, differential DOC assimilation by sponge cells or stable microbiome components may ultimately drive carbon flux in the sponge holobiont.

**Electronic supplementary material:**

The online version of this article (10.1186/s40168-019-0739-x) contains supplementary material, which is available to authorized users.

## Background

Sponges (phylum Porifera) are sessile invertebrates that have long been integral members of benthic communities, appearing in the late Precambrian fossil record (580 million years ago) [1], and contribute to important present-day ecological functions in marine ecosystems. For example, sponges contribute to benthic-pelagic coupling and the mediation of primary production and nitrification via prokaryotic symbionts [2]. Sponges have an extraordinary ability to pump large volumes of water [3–6] and often retain greater than 80% of filtered particulates [7], thus serving the important function of nutrient transfer from pelagic to benthic communities. In coral reef habitats, sponge population densities may be increasing [8], with sponges as abundant as reef-building corals on many Caribbean reefs [9] and expected to play an increasingly important role in coral reef ecology during a period of broad declines in reef habitats [2, 10].

Sponges support rich and intricate microbial communities [11–15] that differ from seawater and sediments [12, 16–19]. The microbial communities of sponges can comprise up to 38% of the total tissue biomass (10^8^–10^10^ bacteria per gram sponge, 2–4 orders of magnitude greater than seawater) in high microbial abundance (HMA) sponges [20, 21]. In low microbial abundance (LMA) sponges, microbial symbionts occur at similar concentrations as seawater (10^6^–10^8^ bacteria cells per gram of sponge) [21], exhibit lower diversity [22], and are generally dominated by *Proteobacteria* [23–25]. The microbial communities of both HMA and LMA sponges are generally specific to each sponge species (even across great distances [26, 27]), with host species explaining 64% of observed beta diversity variation in microbial communities [15] and representing the dominant factor structuring sponge microbial communities for both abundant and rare bacterial taxa [28]. Given the high abundance and host-specificity of microbial communities in sponges, it is suspected that microbes may contribute to carbon and nutrient flux and processing in host sponges.

Recent work suggests that sponges recycle carbon within coral reef ecosystems, consuming dissolved organic matter and excreting particulate matter, a process potentially involving microbial symbionts. This hypothesis, termed the sponge-loop hypothesis [29], is similar to the microbial loop theory proposed by Azam and colleagues [30], in which free-living marine microbes use dissolved organic carbon (DOC), one of the largest organic carbon reservoirs on earth [31], and convert DOC into particulate organic carbon (POC) for consumption by higher trophic levels in pelagic food webs. Similarly, the sponge-loop hypothesis proposes the cycling of DOC into POC, advancing a two-step process by which sponges mediate this conversion: (1) DOC uptake and (2) detritus excretion [29]. Using isotopic tracers, a recent study demonstrated that DOC from coral mucus can be directly transferred into the bulk tissue of the warm-water sponge *Mycale fistulifera* and the cold-water sponge *Hymedesmia coriacea* [32]. Other studies have suggested that DOC exuded by corals [33], algae [33], or seagrasses [34] can be consumed by sponges, and these data provide support for the first component of the sponge-loop hypothesis in different hosts (HMA and LMA) and environments (shallow-water and deep-water reefs). The second component, detritus excretion via rapid cell turnover (cell proliferation and shedding), may not be as widespread a phenomenon as DOC uptake, occurring in cryptic, encrusting sponge species [29, 32, 33], but absent in massive, emergent sponge species [35].

While the role of microbial symbionts in the sponge-loop is unknown, the abundance and composition of microbial symbionts have been shown to affect some aspects of sponge physiology and nutrient cycles. Such patterns most notably occur across the HMA-LMA spectrum, where microbiome distinctions correlate with differences in feeding behaviors. For example, LMA and HMA sponges exhibit different pumping rates, with LMA sponges pumping faster and exhibiting greater choanocyte density than HMA sponges [35–37]. Further, differences in pumping rates, aquiferous system density, and microbial communities between HMA and LMA sponges may also affect host carbon and nutrient flux. In general, HMA sponges exhibit greater uptake rates of DOC than LMA sponges [35, 38, 39] and different inorganic nitrogen fluxes [40]. Indeed, recent work suggests that differential nitrogen cycling between sympatric LMA and HMA sponges results in trophic niche separation, thereby facilitating co-existence and efficient nutrient utilization in oligotrophic environments [41]. The relationships between microbial symbiont communities, DOC flux, and nutrient cycling in coral reef sponges may be important for understanding recent changes to coral reef ecosystems, particularly in the Caribbean [42].

Comparisons of carbon and nutrient flux across the HMA-LMA spectrum have yielded important insights into sponge-mediated nutrient cycling, yet most studies lack comprehensive microbiome characterizations within these broad host categories. Sponge microbiomes differ markedly within the HMA and LMA categories, as most exhibit a species-specific signature [14]. Accordingly, a direct test of the relationship between microbiome composition and sponge nutrient flux has not been conducted. This study investigated whether sponges with different rates of nutrient flux hosted different microbial symbionts, at the community and individual taxa levels, by assessing correlations between DOC, POC, and nutrient flux and the structure and composition of microbial communities in sponges. We characterized the microbiomes of 10 common, emergent (i.e., non-cryptic and non-encrusting) Caribbean coral reef sponge species from two locations to investigate the drivers of inter- and intra-specific variation in sponge microbiomes and determine the relationship between symbiont community variation and carbon and nutrient fluxes.

## Methods

### Sample collection

Ambient seawater and sponge tissue were collected from two geographically distant locations (~ 1203 km apart): Conch Reef, Florida (24° 56.9′ N, 80° 27.2′ W), and Carrie Bow Cay, Belize (16° 48.14′ N, 88° 4.79′ W). Ten of the most common Caribbean coral reef sponges were sampled in Key Largo, Florida (Conch Reef), and eight of these species were sampled in Belize (Carrie Bow Cay) from 13 to 23 m depths in June and July 2016, respectively (Table 1). Only apparently healthy sponge individuals (i.e., no evidence of disease, tissue damage, algal colonization, or epibionts) with a single osculum were sampled (except in the case of *Agelas tubulata* which had multiple oscula) [35]. All sponge tissue samples were collected in separate bags, preserved in 100% ethanol, and stored at − 20 °C until processing. Seawater samples (1 L) were collected at each sampling site and day of sponge tissue sample collection, concentrated onto 0.2 μm filters, preserved in 100% ethanol, and stored at − 20 °C until processing.

**Table 1:** Alpha diversity measurements of microbial communities in 10 sponge species and ambient seawater showing sponge abundance category (HMA vs. LMA) and replicates per location (Conch Reef, Florida, vs. Carrie Bow Cay, Belize). Values are means ± 1 standard error

Sponge Species			Conch Reef, Florida			Carrie Bow Cay, Belize			
	Category	*n*	S	H′	D	*n*	S	H′	D
*Agelas tubulata*	HMA	6	559 ± 22	4.20 ± 0.14	0.035 ± 0.008	6	466 ± 48	3.90 ± 0.14	0.045 ± 0.009
*Aplysina archeri*	HMA	1	288	3.17	0.14	0	–	–	–
*Ircinia strobilina*	HMA	3	505 ± 24	4.53 ± 0.06	0.020 ± 0.003	0	–	–	–
*Verongula gigantea*	HMA	6	534 ± 21	4.55 ± 0.06	0.025 ± 0.002	2	563 ± 68	4.47 ± 0.04	0.027 ± 0.002
*Verongula reiswigi*	HMA	2	533 ± 15	4.73 ± 0.05	0.015 ± 0.001	4	445 ± 36	4.25 ± 0.07	0.028 ± 0.003
*Xestospongia muta*	HMA	5	543 ± 10	4.66 ± 0.02	0.019 ± 0.001	5	545 ± 12	4.53 ± 0.03	0.021 ± 0.001
*Callyspongia plicifera*	LMA	7	716 ± 13*	4.12 ± 0.03	0.042 ± 0.002	5	690 ± 10*	4.04 ± 0.01	0.042 ± 0.005
*Callyspongia vaginalis*	LMA	5	537 ± 33	2.45 ± 0.21	0.323 ± 0.051	5	474 ± 36	2.08 ± 0.30	0.420 ± 0.087
*Mycale laxissima*	LMA	6	433 ± 37	2.27 ± 0.23*	0.250 ± 0.040*	5	324 ± 20	1.59 ± 0.18*	0.409 ± 0.059*
*Niphates digitalis*	LMA	7	503 ± 25	2.33 ± 0.20	0.344 ± 0.053	4	353 ± 36	1.83 ± 0.22	0.419 ± 0.061
Seawater	–	5	661 ± 30	4.04 ± 0.05	0.046 ± 0.001	6	732 ± 18	4.21 ± 0.04	0.037 ± 0.001
Total		53				42			

Asterisks (*) indicate significant within species differences across locations based on Tukey’s HSD tests. *S* OTU richness, *H*′ Shannon-Weaver, and *D* Simpson diversity index

### Sponge barcoding

All ten sponge species represent common Caribbean coral reef species and were identified morphologically following Zea et al. [43]. To confirm identifications made in the field, PCR amplification of the partial mitochondrial cytochrome c oxidase subunit I (COI) gene was amplified using the forward primer LCO1490 and reverse primer HCO2198 for species barcoding [44]. PCR amplification reactions contained 0.5 μl of each primer (10 μM), 12.5 μl (0.5 units) of MyTaq™ Red Mix DNA polymerase (Bioline), 1 μl of DNA template, and PCR water for a total reaction volume of 25 μl. The thermocycler conditions included initial denaturation step (95 °C, 1 min) followed by 35 cycles of denaturation (95 °C, 15 s), annealing (45 °C, 15 s), and extension (72 °C, 10 s), with a final extension step (72 °C, 1 min) and 6 °C hold. The COI amplicons were used in a sequencing PCR with BigDye version 3.1 (Applied Biosystems) and a thermocycler program consisting of an initial denaturation step (96 °C, 1 min), 25 cycles of annealing (50 °C, 5 s), extension (60 °C, 4 min), and denaturation (96 °C, 10 s), followed by a final annealing (50 °C, 5 s), extension (60 °C, 4 min), and 10 °C hold. Amplicons were cleaned using BigDye® XTerminator™ Purification Kit (Thermo Fischer), following the manufacturer’s protocol, and sequenced on an AB 3500 Gene Analyzer (Applied Biosystems) at the UNCW Center for Marine Science. Forward and reverse sequences were aligned in Geneious version 8.1.9 [45] to create consensus sequences and compared to the GenBank database using the nucleotide-nucleotide Basic Local Alignment Search Tool (BLASTn). Sequence data were deposited in GenBank under the accession numbers MH297440 to MH297461.

### Sample metadata

For each sponge tissue sample that was collected, metadata on sponge pumping rates, sponge volumes, and carbon fluxes were collected on in situ colonies (prior to tissue sampling) and processed as reported previously [35]. Briefly, sponge pumping rates were measured using an acoustic Doppler velocimeter (SonTek) [6] and sponge tissue volume was estimated using measurements of the dimensions of each sponge [35]. Paired incurrent (ambient) and excurrent seawater samples (1.5 L) were collected via syringe and subsequently filtered (Whatman GF/F). POC on filters was quantified using a CE Elantech NC2100 elemental analyzer [35, 46], and DOC in the filtrate of each sample was quantified using a Shimadzu TOC 5050 analyzer [35]. In Belize, an additional 40 mL of the filtrate from each incurrent and excurrent seawater sample was collected and stored frozen until quantification of NO_x_, NH_4_, and PO_4_ using a Bran + Luebbe AutoAnalyzer III following standard protocols [47]. The specific filtration rate (SFR, μmol C, or nutrient/s/L sponge) for each carbon and nutrient species (i.e., POC, DOC, NO_x_, NH_4_, and PO_4_) was calculated as:

$$ \mathrm{SFR}=\frac{\left({C}_{\mathrm{in}}-{C}_{\mathrm{ex}}\right)\times Q}{V_{\mathrm{sponge}}} $$

where *C*_in_ and *C*_ex_ are the incurrent and excurrent concentrations of each carbon pool or nutrient type (C/mL), *V*_sponge_ is the sponge tissue volume (L), and *Q* is the pumping rate for each sponge (mL/s); thus, positive values indicate net consumption and negative values indicate net production of a particular carbon pool or nutrient type. Carbon data is available in McMurray et al. [35] and nutrient data in Additional file 1.

### DNA extraction and sequence processing

Ethanol-preserved tissue samples were dissected into 2 mm^3^ cubes that included interior and exterior sponge tissue and were extracted using the DNeasy® Blood & Tissue Kit (Qiagen) following the manufacturer protocols. Partial (V4) 16S rRNA gene sequences were amplified using the 515f forward primer and 806r reverse primer [48] and sequenced on an Illumina MiSeq platform at Molecular Research LP (Shallowater, TX). Illumina sequence reads were processed in mothur v1.38.0 [49] using a modified version of the bioinformatics pipeline described in Weigel and Erwin [17]. Briefly, raw sequences (*n* = 13.9 million) were demultiplexed, quality-filtered, aligned, classified, and clustered into operational taxonomic units (OTUs) at 97% sequence identity (*n*_OTU_ = 25,712). Sequence libraries for each sample were subsampled to the lowest read count (*n* = 15,825), and all data analyses were based on the subsampled dataset. Sequence data were deposited in the Sequence Read Archive of the National Center for Biotechnology under accession number SRP142647.

### Data analysis

#### Microbial community diversity

Diversity statistics (Shannon-Weaver, OTU richness, Simpson) were calculated in mothur using OTU relative abundance data. Two-way nested analyses of variance (ANOVA) were used to test for significant differences in diversity indices for two factors: “source” (sponge species or seawater) and “location” (Key Largo or Belize) nested within source in JMP (version 12.0), followed by Tukey’s honest significant difference (HSD) tests to assess multiple post hoc comparisons of means.

#### Microbial community structure

Bray-Curtis similarity matrices were constructed using square root transformed OTU relative abundance data to give more even representation of rare and abundant taxa in community comparisons using Primer-e (version 6.1.11) and visualized in a cluster dendrogram and a two-dimensional non-metric multidimensional scaling (nMDS) plot. A permutational multivariate analysis of variance (PERMANOVA, version 1.0.1) was conducted to test for significant differences in microbial community structure across two factors: “source” and “location” nested within source, with significance determined by Monte Carlo asymptotic *P* values.

#### OTU-level analyses

Similarity percentage (SIMPER) analysis was used to identify individual OTUs driving the overall dissimilarity between microbial communities within each sponge species, using OTU relative abundance matrices and a cumulative dissimilarity cutoff percentage of 0.70. Significant differences in OTU relative abundances were assessed using Metastats [50] in mothur with 1000 permutations. OTUs of interest were then taxonomically identified and compared to OTUs identified in nutrient correlations (see below).

#### Correlations between microbial community diversity and carbon/nutrient flux

Spearman rank-order correlations were conducted in SigmaPlot (version 11) to assess relationships between microbial diversity indices (Shannon-Weaver, OTU richness, Simpson) and carbon/nutrient flux data (DOC, POC, NO_x_, NH_4_, and PO_4_).

#### Correlations between microbial community structure and carbon/nutrient flux

Distance-based linear models (DistLM) were conducted in Primer-e to assess correlations between microbial community (Bray-Curtis) similarity and carbon/nutrient flux data (DOC, POC, NO_x_, NH_4_, and PO_4_) and visualized with distance-based redundancy analysis (dbRDA) plots. Specifically, carbon and nutrient flux measurements estimated from specific filtration rates (SFR, μmol C, or nutrient/s/L sponge) were tested as marginal predictor variables for microbial community similarity within and among sponge species. Analyses were repeated using Bray-Curtis similarity matrices constructed from untransformed (raw) data, showing identical significance patterns and thus minimal impact of data transformation on statistical results (Additional files 2 and 3). Analyses were also repeated using carbon and nutrient uptake (*C*_in_–*C*_ex_) instead of specific filtration rates, showing similar statistical patterns when considering a metric not influenced by pumping rates (Additional files 4 and 5).

Inter-specific comparisons were conducted at three levels: all sponge species, within each sponge category (HMA, LMA), and between each pairwise species comparison. Pearson correlations were run in JMP to compare nutrient flux data (POC and NH_4_) in the form of SFR and OTU abundance counts for the first 1000 OTUs within each sponge species.

### Functional gene PCR screening

To test for the presence of signature functional genes involved in nitrogen cycling processes (nitrification: ammonia monooxygenase, *amoA/amoB*; nitrogen fixation: nitrogenase, *nifH*; and denitrification: nitrite reductase, *nirS*), the following primer pairs and cited procedures were used: Arch-amoAF and Arch-amoAR [51], AmoBMF and AmoBMR [52], nif1/2 and nif3/4 [53], nirS1F and nirS6R [54]. For all sponge species, at least two replicates were tested for the presence of each functional gene, except for *Aplysina archeri* (only one sample available). If positive, all remaining replicates of that species were tested at both sites. If negative, the PCR was repeated twice for verification.

## Results

### Host species effects (inter-specific variation)

**Fig. 1:**
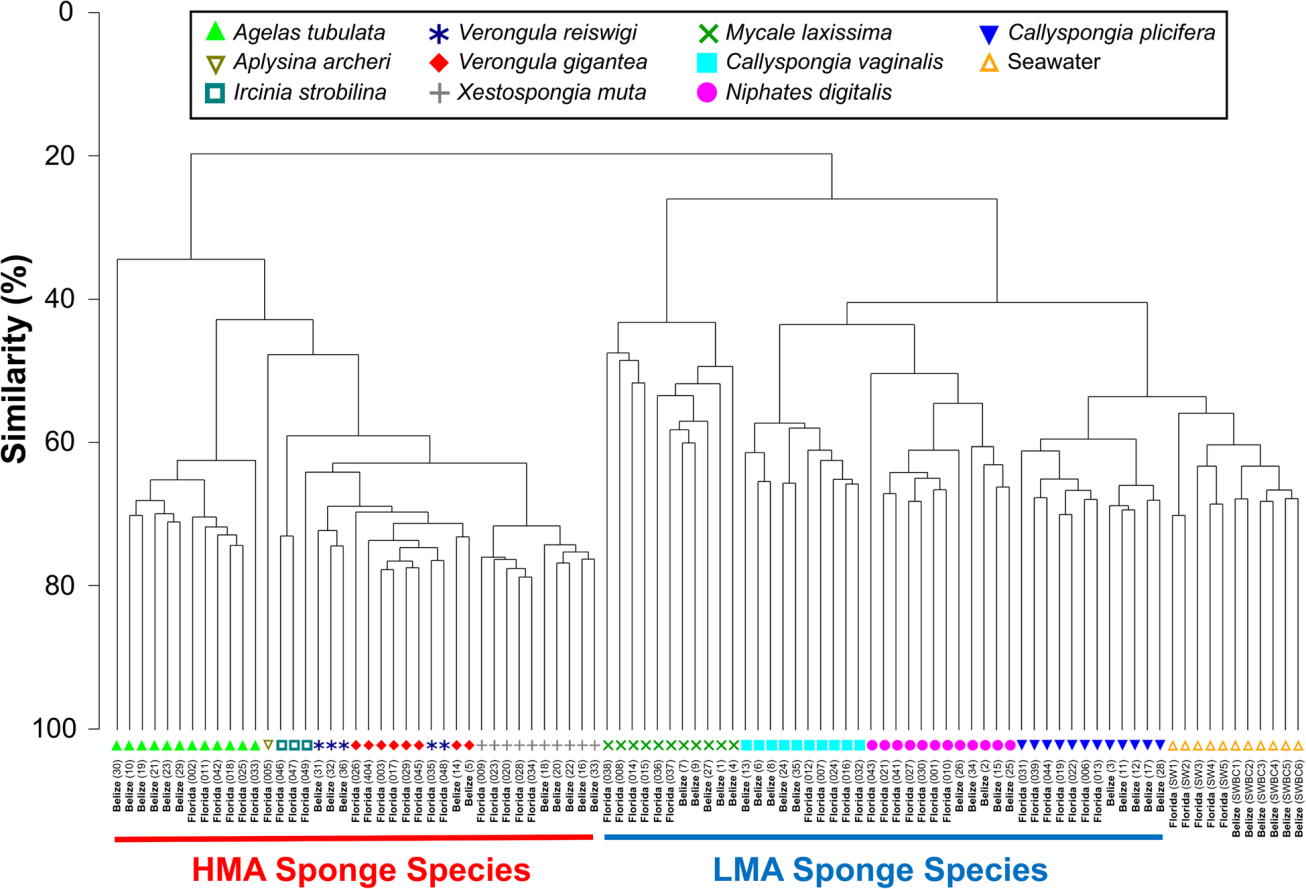
Cluster dendrogram based on Bray-Curtis dissimilarity matrices for all sampled sponge microbial communities collected from both sites. The two main branches separate into HMA (left) and LMA (right) species plus seawater, dividing into respective species (except *V. gigantea* and *V. reiswigi*). All species exhibited some degree of clustering by location (Florida or Belize)

**Fig. 2:**
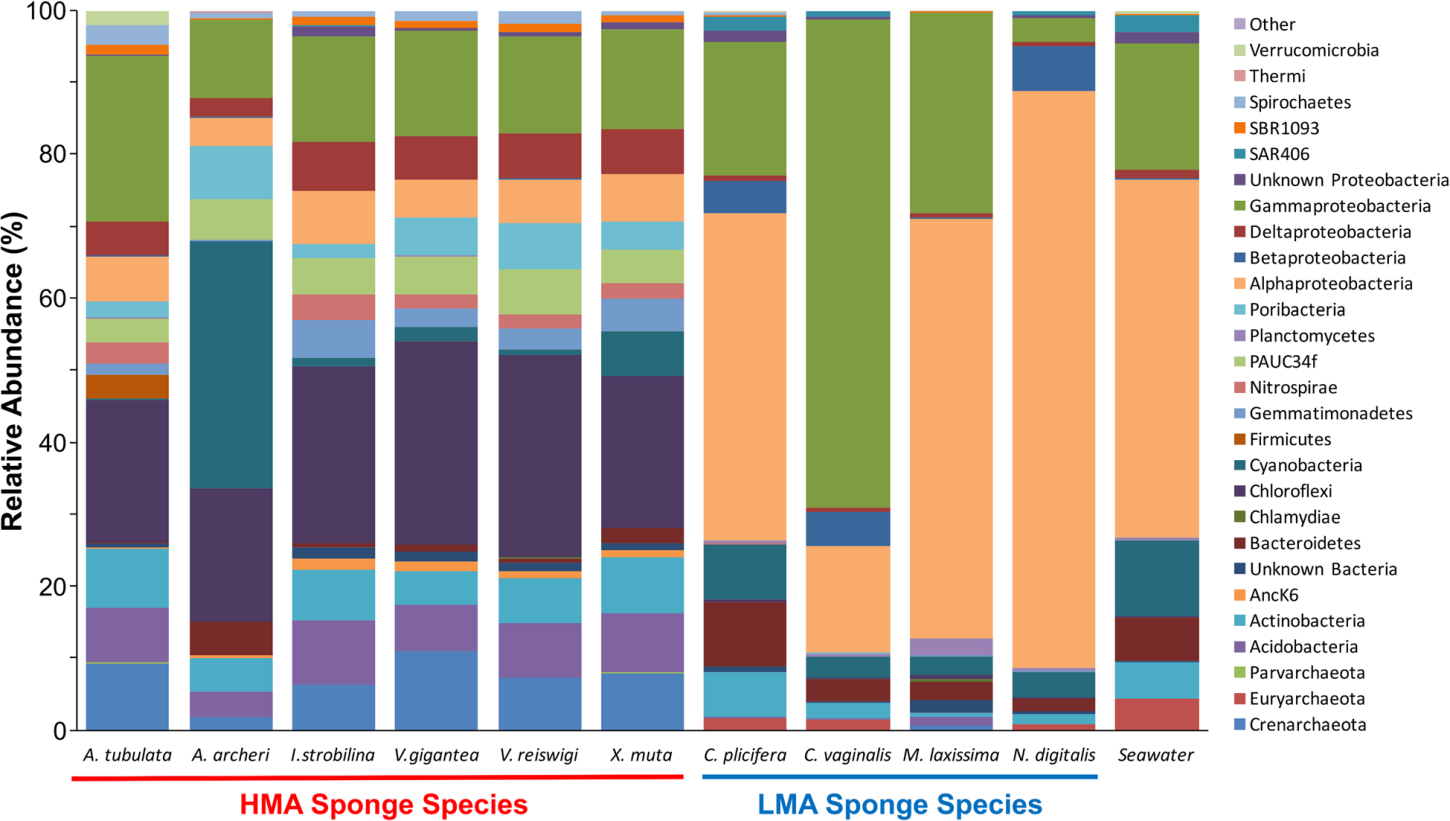
Phylum-level composition of microbial communities of all sampled sponges across sites (Florida and Belize) by host species. The species *A. archeri* is based on the raw count of a single sample, and the species *I. strobilina* is averaged for two samples from the Florida site only (since none were sampled in Belize). Sequences associated with *Proteobacteria* are further divided into class levels

Molecular barcoding confirmed the species identifications from the field (Additional file 6). Microbial communities differed significantly in richness and diversity among sponge species and seawater sources (Shannon-Weaver, *P* < 0.0001; OTU Richness, *P* < 0.0001; Simpson, *P* < 0.0001, Table 1). Similarly, significant differences in microbial community structure were detected among sponge species and seawater sources (*P* = 0.001). A dendrogram based on Bray-Curtis similarity of microbial communities revealed two main branches, one consisting of all HMA sponge species and a second branch consisting of all LMA sponge species plus seawater (Fig. 1). Within each main branch, samples clustered by source (sponge species), except the closely related species *Verongula gigantea* and *V. reiswigi* (Fig. 1). Similar clustering patterns were revealed by nMDS ordination and highlighted a distinct cluster for *Mycale laxissima* within the LMA sponge species (Additional file 7). Differences in microbial community composition were also observed between HMA and LMA sponges, with LMA microbiomes composed primarily of *Alpha*- and *Gammaproteobacteria* and dominated by a small number of OTUs within these taxonomic lineages (Fig. 2). For example, a single OTU (001), an *Alphaproteobacterium* in the genus *Roseivivax*, comprised 60% of all *Niphates digitalis* sequence reads and 77% of sequence reads in *M. laxissima* was affiliated with the orders *Rhodospirillales* (50.4%, class *Alphaproteobacteria*) and *Oceanospirillales* (26.4%, class *Gammaproteobacteria*). In contrast, HMA sponges exhibited a distribution of phyla that was more even, including *Actinobacteria*, *Acidobacteria*, *Chloroflexi*, *Cyanobacteria*, *Poribacteria*, and *Proteobacteria* (Fig. 2).

### Biogeographic effects (intra-specific variation)

Microbial communities differed significantly in richness and diversity among the same sponge species collected from different locations (Shannon-Weaver, *P* = 0.004; richness, *P* < 0.001; Simpson *P* = 0.010). Pairwise tests revealed that these trends were driven by a single species for each metric: only the microbial communities in *M. laxissima* showed significant differences in diversity across locations (higher in Florida; Shannon, *P* = 0.041; Simpson, *P* = 0.005), while OTU richness was only significantly different within microbial communities of *Callyspongia plicifera* between sites (higher in Florida; *P* = 0.007). Accordingly, most species exhibited similar levels of alpha-diversity between locations (Table 1). In contrast, the community structure of sponge microbiomes differed significantly within host species across locations (*P* = 0.001), with three LMA sponge species, one HMA species, and seawater all exhibiting significant differences in microbial community structure between locations: *C. plicifera* (*P* = 0.006), *N. digitalis* (*P* = 0.019), *M. laxissima* (*P* = 0.027), *Xestospongia muta* (*P* = 0.012), and seawater (*P* = 0.024). Further, the Bray-Curtis similarity-based dendrogram revealed that all samples clustered by location (Florida or Belize) within each sponge species or seawater source (Fig. 1).

Individual symbiont OTUs driving these community-level biogeographic trends were determined using similarity percentage (SIMPER) and differential relative abundance (Metastats) analyses. A total of 58 OTUs exhibited significantly different abundances between sampling locations across all sponge species, with nearly all (98%) representing rare members of the microbiome with low individual contributions to community dissimilarity (< 3%, Additional file 8). However, a single OTU (003, *Alphaproteobacteria*) that dominated symbiont communities in *M. laxissima* exhibited significantly higher relative abundance in sponges from Belize (55.2%) than Florida (39.6%) and accounted for > 17% of community dissimilarity between locations. Of these 58 OTUs, 6 were present in 2 or more sponge species, with 1 OTU (006, *Synechococcus*) significantly differing between locations for all LMA sponges (Additional file 8). Additionally, OTU 006 was also detected at significantly higher relative abundances in seawater samples from Florida compared to Belize, comprising 7.2% of sequence reads within Florida seawater samples. Twenty-nine OTUs were found to significantly differ between locations in HMA sponges (7 in A. *tubulata*, 4 in *V. gigantea*, 4 in *V. reiswigi*, and 15 in *X. muta*), and 31 OTUs were found to significantly differ between locations in LMA sponges (15 in *C. plicifera*, 4 in *Callyspongia vaginalis*, 10 in *M. laxissima*, and 8 in *N. digitalis*; Additional file 8). Notably, these OTUs contributed to twice the community dissimilarity in LMA sponges (15%) compared to HMA hosts (7.2%). Nearly one third of these OTUs (*n* = 18, 31%) were affiliated with the phylum *Proteobacteria*, with the majority (*n* = 10, 55%) of proteobacterial OTUs belonging specifically to the class *Gammaproteobacterium*.

### Carbon, nutrient, and microbial abundance correlations

**Fig. 3:**
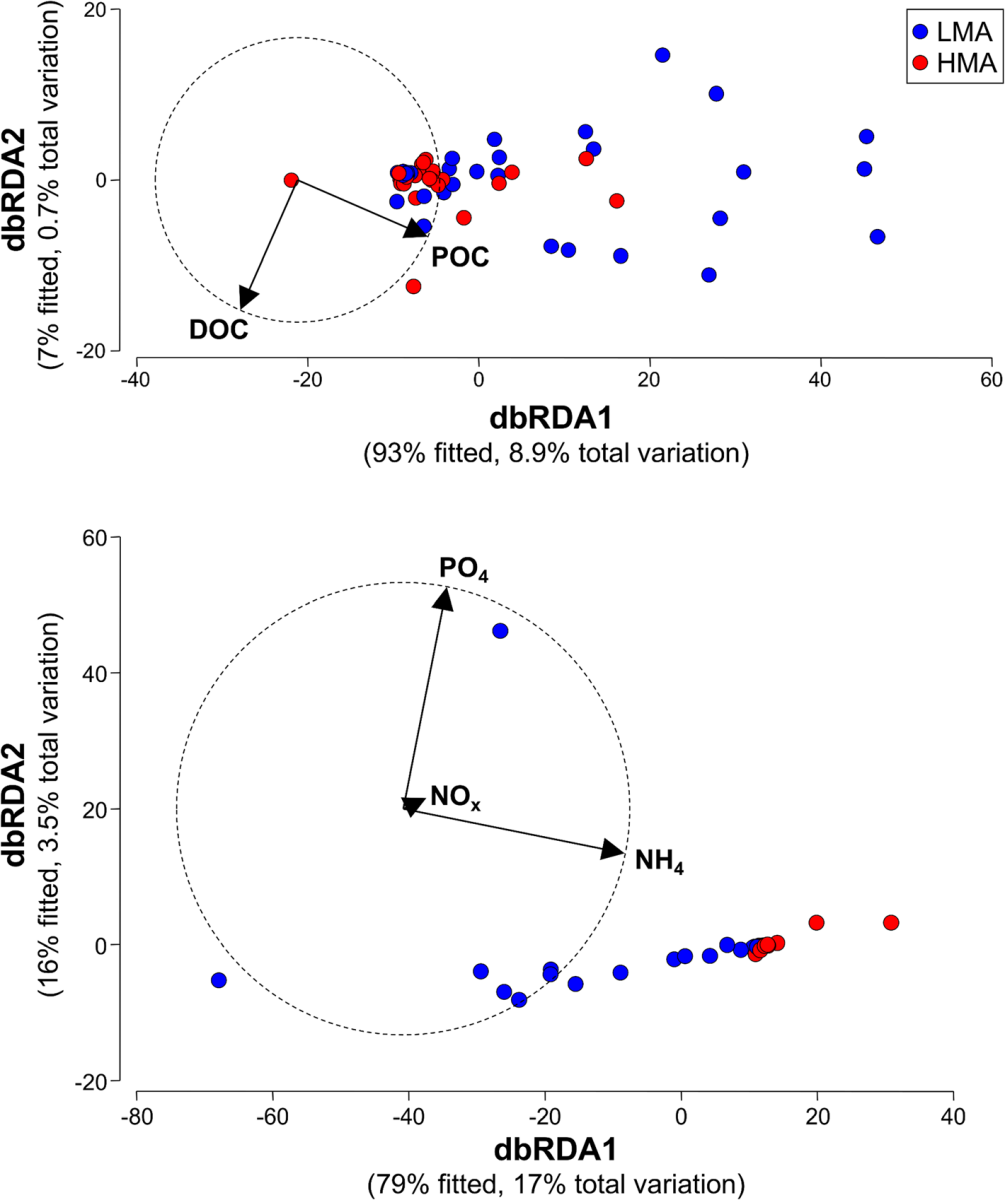
Distance-based redundancy analysis (dbRDA) plot of all sponge samples classified by microbial abundance type (HMA, LMA) and correlated to the carbon/nutrient flux variables, namely DOC and POC specific filtration rates (top) and NH_4_, NO_x_, and PO_4_ specific filtration rates (bottom)

The comparative DistLM analyses showed that microbiome structure varied significantly with POC flux among, but not within, sponge species (Table 2). Significant correlations were detected at inter-specific levels when considering the microbial communities of all host species (*P* = 0.001) and only LMA hosts (*P* = 0.002), while variation in POC flux among HMA hosts did not correlate with variability in microbiome structure (*P* = 0.403, Table 2). Accordingly, pairwise species comparisons were significant for half of the LMA pairs (notably, all involving *M. laxissima*) and most of the LMA-HMA pairs (except those involving *C. vaginalis* and *Verongula* spp.), but none of the HMA pairs (Table 2). In contrast, no significant DistLM correlations were observed between microbiome structure and host DOC flux at any level (inter- and intraspecific, Table 2). No significant correlations were detected with host PO_4_ and NO_x_ flux, but host NH_4_ flux correlated significantly with microbiome structure among sponge species (*P* = 0.002, Table 3). Inter-specific comparisons within LMA and HMA categories were not significant (*P* = 0.073 and 0.549, respectively), with half of the individually paired LMA-HMA sponge species comparisons significant (*P* > 0.05, Table 3). Comparisons of carbon/nutrient flux and microbiome diversity revealed similar patterns: no significant correlations with host DOC, PO_4_, and NO_x_ flux for any alpha-diversity metric, but POC correlated significantly with observed richness (*P* < 0.001) and NH_4_ with Shannon-Weaver and Simpson indices (*P* < 0.001, Additional file 9). Differential carbon and nutrient consumption between LMA and HMA sponges was visualized in dbRDA plots, highlighting the strong correlations between microbiome structure and POC and NH_4_ flux, and the weak correlations with DOC, PO_4_, and NO_x_ (Fig. 3).

**Table 2:** DistLM results correlating POC and DOC specific filtration rates (μmol/s/L sponge) and microbial community structure in sponges from Florida and Belize, showing analysis for all species, by category (HMA/LMA), and for all pairwise species comparisons

Dataset	*P* value		*R* ^2^	
	POC	DOC	POC	DOC
All species	0.001*	0.154	0.084	0.022
All LMA species	0.002*	0.895	0.096	0.015
All HMA species	0.403	0.965	0.033	0.013
Pairwise LMA comparisons				
*M. laxissima–N. digitalis*	0.003*	0.746	0.180	0.046
*M. laxissima–C. plicifera*	0.001*	0.965	0.276	0.020
*M. laxissima–C. vaginalis*	0.002*	0.057	0.105	0.101
*N. digitalis–C. plicifera*	0.289	0.907	0.066	0.033
*N. digitalis–C. vaginalis*	0.477	0.958	0.062	0.038
*C. plicifera–C. vaginalis*	0.167	0.899	0.063	0.027
Pairwise HMA comparisons				
*A. tubulata–V. gigantea*	0.229	0.884	0.089	0.030
*A. tubulata–V. reiswigi*	0.133	0.777	0.116	0.041
*A. tubulata–X. muta*	0.562	0.301	0.041	0.069
*V. gigantea–V. reiswigi*	0.340	0.865	0.099	0.052
*V. gigantea–X. muta*	0.084	0.609	0.126	0.065
*V. reiswigi–X. muta*	0.191	0.695	0.093	0.058
Pairwise LMA-HMA comparisons				
*C. plicifera–A. tubulata*	0.001*	0.586	0.295	0.029
*C. plicifera–V. gigantea*	0.081	0.457	0.138	0.046
*C. plicifera–V. reiswigi*	0.007*	0.795	0.286	0.027
*C. plicifera–X. muta*	0.005*	0.799	0.334	0.019
*M. laxissima–A. tubulata*	0.001*	0.355	0.156	0.055
*M. laxissima–V. gigantea*	0.016*	0.729	0.172	0.044
*M. laxissima–V. reiswigi*	0.605	0.060	0.049	0.134
*M. laxissima–X. muta*	0.001*	0.812	0.144	0.028
*N. digitalis–A. tubulata*	0.020*	0.338	0.181	0.074
*N. digitalis–V. gigantea*	0.348	0.352	0.088	0.094
*N. digitalis–V. reiswigi*	0.082	0.599	0.215	0.072
*N. digitalis–X. muta*	0.035*	0.452	0.228	0.06
*C. vaginalis–A. tubulata*	0.172	0.034	0.077	0.150
*C. vaginalis–V. gigantea*	0.938	0.116	0.023	0.118
*C. vaginalis–V. reiswigi*	0.190	0.135	0.098	0.127
*C. vaginalis–X. muta*	0.131	0.226	0.092	0.081

Asterisks (*) indicate significant P-values

**Table 3:** DistLM results correlating nutrient flux (NH_4_, NO_x_, and PO_4_ specific filtration rates, μmol/s/L sponge) and microbial community structure in sponges from Belize, showing analysis for all species, by category (HMA/LMA) and for all pairwise species comparisons

Dataset	*P* value			*R* ^2^		
	NH_4_	NO_x_	PO_4_	NH_4_	NO_x_	PO_4_
All species	0.002*	0.790	0.153	0.155	0.020	0.043
All LMA species	0.073	0.897	0.178	0.100	0.035	0.084
All HMA species	0.549	0.609	0.933	0.080	0.074	0.037
Pairwise LMA comparisons						
*M. laxissima–N. digitalis*	0.022*	0.718	0.657	0.274	0.104	0.111
*M. laxissima–C. plicifera*	0.032*	0.515	0.095	0.457	0.124	0.315
*M. laxissima–C. vaginalis*	0.040*	0.843	0.696	0.287	0.074	0.081
*N. digitalis–C. plicifera*	0.163	0.981	0.024*	0.189	0.041	0.201
*N. digitalis–C. vaginalis*	0.038*	0.535	0.103	0.261	0.108	0.145
*C. plicifera–C. vaginalis*	0.055	0.278	0.077	0.238	0.123	0.223
Pairwise HMA comparisons						
*A. tubulata–V. gigantea*	0.036*	0.026*	0.043*	0.585	0.582	0.575
*A. tubulata–V. reiswigi*	0.782	0.656	0.239	0.092	0.150	0.282
*A. tubulata–X. muta*	0.871	0.440	0.895	0.050	0.099	0.042
*V. gigantea–V. reiswigi*	0.147	0.360	0.655	0.590	0.582	0.475
*V. gigantea–X. muta*	0.010*	0.125	0.581	0.386	0.399	0.158
*V. reiswigi–X. muta*	0.959	0.226	0.779	0.091	0.185	0.103
Pairwise LMA-HMA comparisons						
*C. plicifera–A. tubulata*	0.014*	0.480	0.046*	0.573	0.125	0.387
*C. plicifera–V. gigantea*	0.123	0.577	0.639	0.479	0.117	0.117
*C. plicifera–V. reiswigi*	0.040*	0.471	0.217	0.522	0.122	0.278
*C. plicifera–X. muta*	0.005*	0.404	0.066	0.624	0.116	0.378
*M. laxissima–A. tubulata*	0.052	0.150	0.397	0.451	0.283	0.123
*M. laxissima–V. gigantea*	0.227	0.126	0.199	0.675	0.677	0.674
*M. laxissima–V. reiswigi*	0.218	0.117	0.201	0.675	0.677	0.674
*M. laxissima–X. muta*	0.416	0.115	0.941	0.155	0.314	0.021
*N. digitalis–A. tubulata*	0.046*	0.895	0.500	0.352	0.098	0.123
*N. digitalis–V. gigantea*	0.002*	0.936	0.507	0.289	0.106	0.125
*N. digitalis–V. reiswigi*	0.149	0.849	0.562	0.299	0.095	0.116
*N. digitalis–X. muta*	0.001*	0.777	0.229	0.389	0.087	0.130
*C. vaginalis–A. tubulata*	0.028*	0.259	0.644	0.360	0.151	0.069
*C. vaginalis–V. gigantea*	0.108	0.077	0.359	0.347	0.572	0.230
*C. vaginalis–V. reiswigi*	0.127	0.656	0.675	0.302	0.087	0.084
*C. vaginalis–X. muta*	0.004*	0.112	0.785	0.407	0.234	0.041

Asterisks (*) indicate significant P-values

In addition to differences at the community level, individual symbiont OTUs that exhibited significant correlations with NH_4_ (*n* = 132, Additional file 10) and POC (*n* = 288, Additional file 11) flux were identified within each host species. Most of these OTUs (> 94%) were rare microbiome members (< 1% relative abundance) with only 11 OTUs also exhibiting significant variation within host species (and where available) across locations (Metastats, *P* < 0.05; Table 4). Of these 11 OTUs, 1 OTU each belonged to *A. tubulata*, *V. gigantea*, and *V. reiswigi*; 3 belonged to *N. digitalis*; and 5 belonged to *X. muta* (Table 4). Only 8 of these 11 OTUs comprised ≥ 1% of average relative abundance in the microbial community of their respective sponge: OTU 060 (*Piscirickettsiaceae*, *V. gigantea*), 070 (*Acidomicrobiales*, *V. reiswigi*), 035 (*Nitrospiraceae*, *X. muta*), 067 (*AncK6*, *X. muta*), 081 (*Chloroflexi*, *X. muta*), 090 (*Cenarchaeum*, *A. tubulata*), and 011 (*Alphaproteobacteria*, *N. digitalis*; Table 4).

**Table 4:** Taxonomy of OTUs that exhibited relative abundances significantly correlated with NH_4_ or POC specific filtration rates (Pearson) and differed significantly within sponge species across locations (Metastats)

Species	OTU	Phylum	Lowest taxonomy	Correlation source	Pearson correlation	Correlation *P* value	Metastats *P* value	Ave. relative Abd.	
								Florida	Belize
*A. tubulata*	090	*Crenarchaeota*	G*. Cenarchaeum*	NH_4_	0.9974	0.0026	0.0506	2.3	1.1
*N. digitalis*	011	*Proteobacterium*	C. *Alphaproteobacterium*	NH_4_	0.9379	0.0184	0.0275	1.3	0.5
	028	*Proteobacterium*	G. *Candidatus* Portiera	NH_4_	0.8830	0.0472	0.0320	0.5	0.2
	045	*Euryarchaeota*	C. *Thermoplasmata*	NH_4_	0.9041	0.0351	0.0114	0.6	0.1
*V. gigantea*	060	*Proteobacterium*	G. *Piscirickettsiaceae*	POC	0.7149	0.0463	0.0226	1.24	0.51
*V. reiswigi*	070	*Acidobacteria*	C. *BPC015*	POC	− 0.8370	0.0377	0.0044	1.78	0.45
*X. muta*	035	*Nitrospirae*	O. *Nitrospiraceae*	POC	0.6880	0.0279	0.0179	2.25	1.61
	067	*AncK6*	P. *AncK6*	POC	− 0.6387	0.0469	0.0600	0.96	1.13
	081	*Chloroflexi*	C. *TK18*	POC	− 0.6472	0.0431	0.0033	1.54	2.89
	129	*Proteobacterium*	F. *Rhodospirillaceae*	POC	0.8114	0.0044	0.0524	0.45	0.30
	134	*Chloroflexi*	C. *mle1-48*	POC	0.6884	0.0277	0.0137	0.04	0.39

*P* phylum, *C* class, *O* order, *F* family, *G* genus

### Functional gene PCR screening

Archaeal ammonia monooxygenase (*amoA*) genes were successfully amplified in nearly all HMA sponge species (*A. tubulata*, *V. gigantea*, *V. reiswigi*, and *X. muta*), while nitrite reductase (*nirS*) genes were only amplified in *A. tubulata* (Florida and Belize) and *V. reiswigi* (Belize, Table 5). Nitrogenase (*nifH*) and bacterial ammonia monooxygenase (*amoB*) genes were not successfully amplified in any samples, despite triplicate reactions attempted.

**Table 5:** PCR-based screening of nitrogen cycling genes in 10 sponge species from 2 locations, showing the number of samples testing positive (left value) and the total number of samples tested (right value) for 4 functional genes. Amplifications for *amoB* and *nifH* genes were repeated in triplicate for each sample

Category	Species	Location	Function gene PCR assays			
			*amoA*	*nirS*	*amoB*	*nifH*
HMA	*A. archeri*	Florida	0/1	0/1	0/1	0/1
	*A. tubulata*	Florida	6/6	5/5	0/1	0/1
		Belize	4/5	3/4	0/1	0/1
	*I. strobilina*	Florida	0/2	0/2	0/2	0/2
	*V. gigantea*	Florida	4/6	0/2	0/1	0/1
		Belize	2/2	0/2	0/1	0/1
	*V. reiswigi*	Florida	1/2	0/2	0/1	0/1
		Belize	2/4	1/4	0/1	0/1
	*X. muta*	Florida	5/5	0/5	0/1	0/1
		Belize	4/5	0/5	0/1	0/1
LMA	*C. plicifera*	Florida	0/3	0/2	0/1	0/1
		Belize	0/3	0/2	0/1	0/1
	*C. vaginalis*	Florida	0/3	0/2	0/1	0/1
		Belize	0/3	0/2	0/1	0/1
	*M. laxissima*	Florida	0/3	0/2	0/1	0/1
		Belize	0/3	0/2	0/1	0/1
	*N. digitalis*	Florida	0/3	0/2	0/1	0/1
		Belize	0/3	0/2	0/1	0/1

## Discussion

Matching emerging trends in the field of sponge microbiology, the microbial communities in 10 common Caribbean sponge species were significantly different from seawater [16, 17] and exhibited a high degree of host specificity [16, 26], with greater intra- than interspecific similarity across locations [28, 37, 55]. Significant differences in diversity and composition between microbial communities of HMA and LMA sponges were also observed, with LMA sponge microbial communities exhibiting lower diversity and higher relative abundances of *Proteobacteria*, consistent with previous work [22]. One LMA sponge species, *M. laxissima*, clustered separately from other LMA hosts and was recently reported to exhibit slower pumping rates and differential nutrient utilization compared to other LMA sponge species from the same Caribbean sites [35]. In addition, a significant effect of location on microbial diversity and composition was detected within each host sponge species, indicating small intra-specific shifts in microbial communities across sites. These biogeographic differences were primarily manifested in beta-diversity metrics (i.e., compositional differences), although significant differences in alpha-diversity were observed for two LMA hosts (richness in *C. plicifera* and Shannon-Weaver index in *M. laxissima*). Accordingly, our microbiome dataset encompassed variability among and within sponge species and allowed for correlations at multiple levels between variability in microbiome structure and variation in carbon and nutrient fluxes.

Surprisingly, no significant correlations between symbiont structure and DOC flux were detected among or within any sponge species. POC flux did track with symbiont structure across the HMA-LMA spectrum but explained little of the variation in POC flux among sponge species within these categories and none of the variation within sponge species. Matching these findings, individual OTUs found to correlate with POC flux were mostly rare (< 1% relative abundance), with only four OTUs comprising > 1% relative abundance. Together, these results indicate that microbial communities do not exhibit clear and consistent structural shifts with variable rates of holobiont carbon flux and thus do not explain variability in DOC uptake among sponges as hypothesized previously [35, 39]. These findings may indicate that sponge cells, not microbial cells, are the primary site of DOC uptake, as suggested by recent experiments tracing DOC incorporation into bacteria-specific and sponge-specific phospholipid-derived fatty acids [33]. Alternatively, DOC uptake may only occur in a specific portion of the sponge microbiome (e.g., stable or functionally redundant taxa), thereby dissociating overall symbiont structure and carbon cycling. Indeed, recent genomic evidence from sponge symbiont phyla enriched in HMA sponges revealed a complex suite of carbohydrate-degrading genes [56, 57]. In either case, our results suggest that divergent physiologies and symbiont abundances across the HMA-LMA spectrum play a greater role in DOC uptake than the fine-scale (OTU level) composition of the microbial consortia in any given species. Compositional insights from amplicon sequence data represent an important first step in characterizing microbiomes and assessing the impacts of environmental factors on microbiome structure; however, there are also technical limitations associated with the nature of relative abundance data [58]. Thus, correlations between absolute symbiont abundance and DOC uptake may not be detected by the methods employed herein and remain a target for future study.

In contrast to the observed decoupling of symbiont structure and carbon flux, microbial community structure correlated strongly with NH_4_ flux across the HMA-LMA spectrum. Previous work has shown greater NH_4_ consumption by HMA than LMA sponges [34, 40, 41] and different nitrogen trophic levels for Caribbean HMA versus LMA sponges [59], indicating differential nitrogen cycling pathways in HMA and LMA sponge microbiomes. Our results show that variations in NH_4_ flux between HMA and LMA sponge hosts specifically track with microbiome composition and suggest that nitrification is a key energy generation process in the sponge microbiome with cascading effects on the entire prokaryotic symbiont community. Supporting these conclusions, symbiont taxa affiliated with ammonia-oxidizing (*Thaumarchaeota* [60]) and nitrite-oxidizing (*Nitrospirae*) lineages were more common (an order of magnitude greater relative abundance) in HMA compared to LMA microbiomes. Further, we identified individual symbiont OTUs that correlated with NH_4_ flux, including the archaeal OTU-090 (*Thaumarchaeota*) in the HMA host *A. tubulata* whose relative abundance increased with greater levels of NH_4_ uptake. In general, prokaryotic functional guilds involved in nitrogen cycling are restricted to specific taxonomic groups, whereas DOC uptake is a general process common to nearly all prokaryotic taxa. Thus, the strong link between symbiont structure and nitrogen cycling observed among the holobionts of the sponges investigated herein, and the weak link with carbon cycling, may result from the narrow phylogenetic distribution of functional guilds in the nitrogen cycle.

The presence and expression of functional genes encoding for key metabolic enzymes offers additional insight into nutrient cycling within the sponge microbiome. Herein, we detected ammonia monooxygenase (*amoA*) genes in most HMA sponges (four of six species), supporting the presence of nitrifying symbionts that use ammonia as a substrate for energy generation. Functional gene screening also revealed the presence of nitrite reductase (*nirS*) genes in two HMA sponge species, indicating the potential for denitrification pathways in some sponge microbiomes. Indeed, sponge microbial communities have been shown to contain members capable of numerous nitrogen transformation pathways, including nitrogen fixation, denitrification, and nitrification [53]. Notably, our study did not detect nitrogen-fixing bacterial taxa or *nifH* genes in Caribbean sponges, despite their previous detection in the same sponge species (*Ircinia strobilina* and *M. laxissima*) from the same site (Key Largo, Florida) [53], and primers targeting *amoB* gene subunits were negative, despite amplification of *amoA* gene subunits. Technical reasons may account for the lack of gene detection herein, and further metagenomic studies are required to confirm these results. Similar PCR screening for genes involved in carbon flux (e.g., DOC transporters) is complicated by the vast diversity of these genes, but is now approachable using metagenomic (or metatranscriptomic) techniques (e.g., Poretsky et al. [61]). Such approaches to symbiont characterization will aid in clarifying the presence and activity of symbiont functional guilds in the sponge microbiome, as well as the relationship between symbiont *activity* and carbon and nutrient flux.

## Conclusions

Our results reveal novel insights into the relationship between symbiont structure and holobiont carbon and nutrient flux, while also confirming previous findings regarding the drivers of sponge microbial community structure and differences across the HMA-LMA spectrum. Our results show sponge microbial communities are structured by host species and, to a lesser degree, biogeography, yet inter- and intra-specific variation in sponge microbiomes are uncoupled from sponge carbon flux. As such, these results do not support previous theories that variations in microbial community structure are specifically related to differential DOC flux in host sponges [39, 62, 63]. Rather, the relationship between ambient DOC, seawater flux, and the thresholds in DOC flux previously observed [38, 41, 46] is likely due to differential assimilation by sponge cells [33, 64] or is mediated by stable members of sponge microbiomes. Future research assessing the differential regulation of genes involved in DOC uptake (e.g., DOC transporters) may provide additional insight into the physiology of stable microbiome members and cascading effects on holobiont carbon flux. In contrast to carbon flux, the correlational and functional gene data support previous findings of sponge microbial communities having the ability to participate in nitrogen cycling [65–67] and implicate nitrification as a key metabolic process affecting overall microbiome structure in Caribbean sponges [40]. Together, these results indicate that the sponge microbiome may play important roles in nutrient cycling within coral reef ecosystems and should be considered when assessing the ecological impacts of sponges on reef communities, including nutrient feedback loops causing ecosystem phase shifts on coral reefs.

## Additional files

Additional file 1: Sponge volumes, pumping rates, nutrient uptake, and nutrient flux data from Belize sponge holobionts used in DistLM analyses. (DOCX 19 kb)

Additional file 2: Comparison of DistLM results using Bray-Curtis similarity based on square-root transformed vs. untransformed (raw) data, showing analysis for all species and by category (HMA/LMA). (DOCX 15 kb)

Additional file 3: Comparison of DistLM results using Bray-Curtis similarity based on square-root transformed vs. untransformed (raw) data, showing analysis for all species and by category (HMA/LMA). (DOCX 15 kb)

Additional file 4: Comparison of DistLM results using POC and DOC flux (specific filtration rates, SFR) vs. POC and DOC uptake (*C*_*in*_ – *C*_*ex*_, In-Ex), showing analysis for all species and by category (HMA/LMA). (DOCX 14 kb)

Additional file 5: Comparison of DistLM results using nutrient flux (specific filtration rates, SFR) vs. nutrient uptake (*C*_*in*_ – *C*_*ex*,_ In-Ex), showing analysis for all species and by category (HMA/LMA). (DOCX 15 kb)

Additional file 6: Field (morphology-based) species identifications and top BLASTn species identifications from amplified barcoding sequences. (DOCX 14 kb)

Additional file 7: Bray-Curtis dissimilarity matrices based 2D NMDs cluster plot of microbial communities for all samples (sponge and seawater) across locations (Belize and Florida). (DOCX 78 kb)

Additional file 8: Taxonomic classifications and top BLASTn matches (sources in parenthesis, sponge species names in italics) of common microbial OTUs between locations (Belize and Florida). (DOCX 38 kb)

Additional file 9: Spearman (rank order) correlations between carbon (DOC, POC) and nutrient flux (NH_4_, NO_x_, and PO_4_ specific filtration rates, μmol/s/L sponge) and microbial community (alpha) diversity metrics, showing correlation coefficients (*R*) and significance (*P*) values. (DOCX 20 kb)

Additional file 10: Highly correlated microbial abundances and NH_4_ specific filtration rates for all sponges with more than 2 individuals sampled in Belize with a significant *P*-value (< 0.05). (DOCX 33 kb)

Additional file 11: Significant differences in correlated microbial abundance and POC specific filtration rates for all sponges sampled at both sites that had more than 2 individuals sampled at each site and a significant *P*-value (< 0.05). (DOCX 56 kb)

## Data Availability

The 16S rRNA sequence datasets generated and analyzed in the current paper are available in the Sequence Read Archives (SRP142647) May 31, 2019, at the following link https://www.ncbi.nlm.nih.gov/sra/SRP142647. The host sequences generated in the current study have been submitted to GenBank under the title “Microbial Symbionts, Carbon and Nutrient Cycling in Caribbean Coral Reef Sponges”.
