# Supplementary material for: Testing the relationship between microbiome composition and flux of carbon and nutrients in Caribbean coral reef sponges
Source: Microbiome. 2019 Aug 29;7:124. doi: 10.1186/s40168-019-0739-x (PMC6716902; doi:10.1186/s40168-019-0739-x)
Supplement: Supplementary file 1 — Sponge volumes, pumping rates, nutrient uptake, and nutrient flux data from Belize sponge holobionts used in DistLM analyses. (DOCX 19 kb) [file 40168_2019_739_MOESM1_ESM.docx]

**Additional file 1.** Sponge volumes, pumping rates, nutrient uptake, and nutrient flux data from Belize sponge holobionts used in DistLM analyses. (DOCX)

|  |  |  | **Pump Rate** | **Sponge Volume** | **Uptake** (μM In – μM Ex) | | | **Specific Filtration Rate** (μmol s^-1^ L^-1^) | | |
| --- | --- | --- | --- | --- | --- | --- | --- | --- | --- | --- |
| **Sample** | **Category** | **Species** | (mL/s) | (mL) | PO_4_ | NO_x_ | NH_4_ | PO_4_ | NO_x_ | NH_4_ |
| Belize.10 | HMA | *Agelas tubulata* | 43.105 | 5507 | -0.015 | -1.160 | 0.055 | -0.000117 | -0.009079 | 0.000430 |
| Belize.19 | HMA | *Agelas tubulata* | 11.092 | 1417 | -0.015 | -0.590 | 0.020 | -0.000117 | -0.004618 | 0.000157 |
| Belize.21 | HMA | *Agelas tubulata* | 1.319 | 1196 | -0.010 | -1.690 | -0.360 | -0.000011 | -0.001863 | -0.000397 |
| Belize.23 | HMA | *Agelas tubulata* | 6.534 | 3321 | -0.015 | -0.320 | 0.005 | -0.000030 | -0.000630 | 0.000010 |
| Belize.5 | HMA | *Verongula gigantea* | 341.730 | 6208 | -0.010 | -0.940 | 0.100 | -0.000550 | -0.051742 | 0.005505 |
| Belize.31 | HMA | *Verongula reiswigi* | 15.126 | 5451 | -0.110 | -0.845 | -0.005 | -0.000305 | -0.002345 | -0.000014 |
| Belize.32 | HMA | *Verongula reiswigi* | 17.533 | 6077 | -0.020 | -0.470 | 0.055 | -0.000058 | -0.001356 | 0.000159 |
| Belize.16 | HMA | *Xestospongia muta* | 651.111 | 59371 | 0.070 | -1.070 | 0.205 | 0.000768 | -0.011735 | 0.002248 |
| Belize.18 | HMA | *Xestospongia muta* | 100.875 | 43050 | -0.030 | -1.715 | 0.025 | -0.000070 | -0.004019 | 0.000059 |
| Belize.20 | HMA | *Xestospongia muta* | 99.134 | 56475 | -0.010 | -0.465 | 0.025 | -0.000018 | -0.000816 | 0.000044 |
| Belize.22 | HMA | *Xestospongia muta* | 296.167 | 16882 | -0.040 | -0.755 | -0.060 | -0.000702 | -0.013245 | -0.001053 |
| Belize.33 | HMA | *Xestospongia muta* | 132.481 | 24171 | -0.010 | -0.770 | 0.015 | -0.000055 | -0.004220 | 0.000082 |
| Belize.3 | LMA | *Callyspongia plicifera* | 65.766 | 453 | -0.005 | 0.030 | -0.130 | -0.000726 | 0.004358 | -0.018883 |
| Belize.11 | LMA | *Callyspongia plicifera* | 91.463 | 1039 | -0.020 | -0.215 | -0.215 | -0.001760 | -0.018924 | -0.018924 |
| Belize.12 | LMA | *Callyspongia plicifera* | 89.191 | 834 | -0.010 | -0.100 | -0.135 | -0.001070 | -0.010698 | -0.014443 |
| Belize.17 | LMA | *Callyspongia plicifera* | 77.064 | 1714 | 0.000 | 0.030 | -0.130 | 0.000000 | 0.001349 | -0.005845 |
| Belize.28 | LMA | *Callyspongia plicifera* | 28.843 | 330 | -0.020 | -1.230 | -0.290 | -0.001749 | -0.107550 | -0.025357 |
| Belize.6 | LMA | *Callyspongia vaginalis* | 84.5110 | 2029 | -0.015 | -0.115 | -0.260 | -0.000625 | -0.004790 | -0.010830 |
| Belize.8 | LMA | *Callyspongia vaginalis* | 74.529 | 1389 | -0.005 | -0.130 | -0.135 | -0.000268 | -0.006974 | -0.007242 |
| Belize.13 | LMA | *Callyspongia vaginalis* | 4.687 | 834 | -0.015 | 0.000 | -0.275 | -0.000084 | 0.000000 | -0.001546 |
| Belize.24 | LMA | *Callyspongia vaginalis* | 133.985 | 3586 | 0.010 | 0.080 | -0.075 | 0.000374 | 0.002989 | -0.002802 |
| Belize.35 | LMA | *Callyspongia vaginalis* | 93.245 | 639 | 0.000 | 0.030 | -0.110 | 0.000000 | 0.004379 | -0.016055 |
| Belize.4 | LMA | *Mycale laxissima* | 3.446 | 1359 | -0.010 | -0.150 | -0.240 | -0.000025 | -0.000380 | -0.000609 |
| Belize.9 | LMA | *Mycale laxissima* | 2.537 | 1001 | -0.025 | -0.180 | -0.270 | -0.000063 | -0.000456 | -0.000685 |
| Belize.27 | LMA | *Mycale laxissima* | 5.496 | 2167 | -0.015 | -0.125 | -0.140 | -0.000038 | -0.000317 | -0.000355 |
| Belize.2 | LMA | *Niphates digitalis* | 329.064 | 1537 | 0.100 | -0.050 | -0.210 | 0.021404 | -0.010702 | -0.044948 |
| Belize.15 | LMA | *Niphates digitalis* | 27.493 | 1094 | -0.010 | -0.100 | -0.160 | -0.000251 | -0.002514 | -0.004022 |
| Belize.25 | LMA | *Niphates digitalis* | 30.835 | 515 | -0.020 | -1.695 | -0.515 | -0.001197 | -0.101452 | -0.030825 |
| Belize.26 | LMA | *Niphates digitalis* | 4.096 | 3117 | -0.010 | 0.770 | -0.090 | -0.000013 | 0.001012 | -0.000118 |
| Belize.34 | LMA | *Niphates digitalis* | 290.822 | 2690 | 0.020 | -0.010 | -0.415 | 0.002162 | -0.001081 | -0.044863 |
