# Supplementary material for: Testing the relationship between microbiome composition and flux of carbon and nutrients in Caribbean coral reef sponges
Source: Microbiome. 2019 Aug 29;7:124. doi: 10.1186/s40168-019-0739-x (PMC6716902; doi:10.1186/s40168-019-0739-x)
Supplement: Supplementary file 2 — Comparison of DistLM results using Bray-Curtis similarity based on square-root transformed vs. untransformed (raw) data, showing analysis for all species and by category (HMA/LMA). (DOCX 15 kb) [file 40168_2019_739_MOESM2_ESM.docx]

**Additional file 3.** Comparison of DistLM results using Bray-Curtis similarity based on square-root transformed vs. untransformed (raw) data, showing analysis for all species and by category (HMA/LMA). Asterisks (*) highlight significant outcomes (*P* < 0.05). (DOCX)

|  |  | **P** | | | **R^2^** | | |
| --- | --- | --- | --- | --- | --- | --- | --- |
| **Comparison** | Data | NH_4_ | NO_x_ | PO_4_ | NH_4_ | NO_x_ | PO_4_ |
| All Species | Transformed (√) | 0.002* | 0.790 | 0.153 | 0.155 | 0.020 | 0.043 |
|  | Raw | 0.001* | 0.838 | 0.106 | 0.150 | 0.019 | 0.046 |
| All LMA | Transformed (√) | 0.073 | 0.897 | 0.178 | 0.100 | 0.035 | 0.084 |
|  | Raw | 0.070 | 0.787 | 0.145 | 0.127 | 0.033 | 0.089 |
| All HMA | Transformed (√) | 0.549 | 0.609 | 0.933 | 0.080 | 0.074 | 0.037 |
|  | Raw | 0.576 | 0.664 | 0.949 | 0.069 | 0.062 | 0.022 |
