# Supplementary material for: Testing the relationship between microbiome composition and flux of carbon and nutrients in Caribbean coral reef sponges
Source: Microbiome. 2019 Aug 29;7:124. doi: 10.1186/s40168-019-0739-x (PMC6716902; doi:10.1186/s40168-019-0739-x)
Supplement: Supplementary file 4 — Comparison of DistLM results using POC and DOC flux (specific filtration rates, SFR) vs. POC and DOC uptake (Cin – Cex, In-Ex), showing analysis for all species and by category (HMA/LMA). (DOCX 14 kb) [file 40168_2019_739_MOESM4_ESM.docx]

**Additional file 4.** Comparison of DistLM results using POC and DOC flux (specific filtration rates, SFR) vs. POC and DOC uptake (*C_in_* – *C_ex_*, In-Ex), showing analysis for all species and by category (HMA/LMA). Asterisks (*) highlight significant outcomes (*P* < 0.05). (DOCX)

|  |  | **P** | | **R^2^** | |
| --- | --- | --- | --- | --- | --- |
| **Comparison** | **Data** | POC | DOC | POC | DOC |
| All Species | SFR | 0.001* | 0.154 | 0.084 | 0.022 |
|  | In-Ex | 0.100 | 0.141 | 0.025 | 0.022 |
| All LMA | SFR | 0.002* | 0.895 | 0.096 | 0.015 |
|  | In-Ex | 0.012* | 0.948 | 0.066 | 0.013 |
| All HMA | SFR | 0.403 | 0.965 | 0.033 | 0.013 |
|  | In-Ex | 0.045* | 0.325 | 0.070 | 0.035 |
