# Supplementary material for: Testing the relationship between microbiome composition and flux of carbon and nutrients in Caribbean coral reef sponges
Source: Microbiome. 2019 Aug 29;7:124. doi: 10.1186/s40168-019-0739-x (PMC6716902; doi:10.1186/s40168-019-0739-x)
Supplement: Supplementary file 5 — Comparison of DistLM results using nutrient flux (specific filtration rates, SFR) vs. nutrient uptake (Cin – Cex, In-Ex), showing analysis for all species and by category (HMA/LMA). (DOCX 15 kb) [file 40168_2019_739_MOESM5_ESM.docx]

**Additional file 5.** Comparison of DistLM results using nutrient flux (specific filtration rates, SFR) vs. nutrient uptake (*C_in_* – *C_ex_*_,_ In-Ex), showing analysis for all species and by category (HMA/LMA). Asterisks (*) highlight significant outcomes (*P* < 0.05). (DOCX)

|  |  | **P** | | | **R^2^** | | |
| --- | --- | --- | --- | --- | --- | --- | --- |
| **Comparison** | **Data** | NH_4_ | NO_x_ | PO_4_ | NH_4_ | NO_x_ | PO_4_ |
| All Species | SFR | 0.002* | 0.790 | 0.153 | 0.155 | 0.020 | 0.043 |
|  | In-Ex | 0.001* | 0.004* | 0.250 | 0.199 | 0.142 | 0.045 |
| All LMA | SFR | 0.073 | 0.897 | 0.178 | 0.100 | 0.035 | 0.084 |
|  | In-Ex | 0.140 | 0.998 | 0.197 | 0.088 | 0.020 | 0.079 |
| All HMA | SFR | 0.549 | 0.609 | 0.933 | 0.080 | 0.074 | 0.037 |
|  | In-Ex | 0.205 | 0.936 | 0.576 | 0.131 | 0.040 | 0.069 |
