# Supplementary material for: Testing the relationship between microbiome composition and flux of carbon and nutrients in Caribbean coral reef sponges
Source: Microbiome. 2019 Aug 29;7:124. doi: 10.1186/s40168-019-0739-x (PMC6716902; doi:10.1186/s40168-019-0739-x)
Supplement: Supplementary file 6 — Field (morphology-based) species identifications and top BLASTn species identifications from amplified barcoding sequences. (DOCX 14 kb) [file 40168_2019_739_MOESM6_ESM.docx]

**Additional file 6.** Field (morphology-based) species identifications and top BLASTn species identifications from amplified barcoding sequences. n = number of barcode sequences matched to reference sequence. (DOCX)

| Original Species ID | n | Top BLASTn Match | Accession Number | % Identity |
| --- | --- | --- | --- | --- |
| *Agelas tubulata* | 6 | *Agelas conifera* | DQ075712 | 99.7-100 |
| *Agelas tubulata* | 1 | *Agelas* sp. | AY561929 | 95.8 |
| *Niphates digitalis* | 2 | *Niphates digitalis* | EF519655 | 99.3-99.8 |
| *Verongula gigantea* | 3 | *Verongula gigantea* | AM076984 | 99.4-100 |
| *Verongula reiswigi* | 1 | *Verongula reiswigi* | KT921334 | 99.7 |
| *Xestospongia muta* | 7 | *Xestospongia muta* | HQ452958 | 98-100 |
| *Xestospongia muta* | 1 | *Xestospongia muta* | EF519697 | 100 |
