# Supplementary material for: Testing the relationship between microbiome composition and flux of carbon and nutrients in Caribbean coral reef sponges
Source: Microbiome. 2019 Aug 29;7:124. doi: 10.1186/s40168-019-0739-x (PMC6716902; doi:10.1186/s40168-019-0739-x)
Supplement: Supplementary file 7 — Bray-Curtis dissimilarity matrices based 2D NMDs cluster plot of microbial communities for all samples (sponge and seawater) across locations (Belize and Florida). (DOCX 78 kb) [file 40168_2019_739_MOESM7_ESM.docx]

**Additional file 7.** Bray-Curtis dissimilarity matrices based 2D NMDs cluster plot of microbial communities for all samples (sponge and seawater) across locations (Belize and Florida). Three clusters observed are LMA sponges and seawater, *M. laxissima*, and HMA sponges. (DOCX)

**
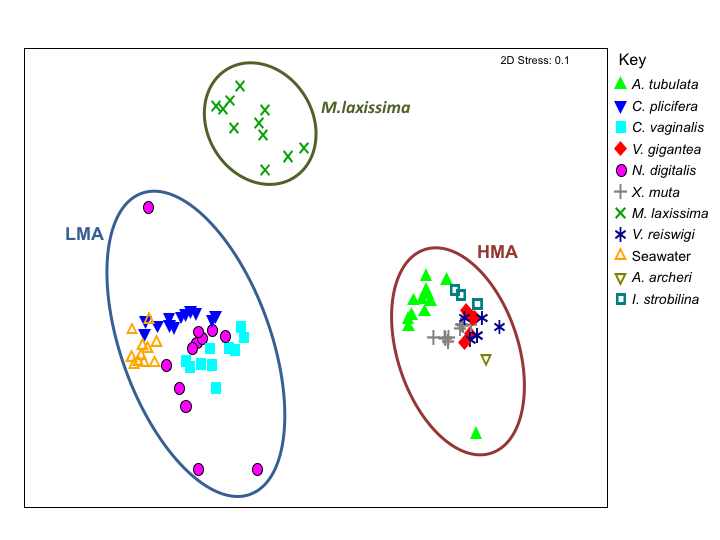
**
