# Supplementary material for: Testing the relationship between microbiome composition and flux of carbon and nutrients in Caribbean coral reef sponges
Source: Microbiome. 2019 Aug 29;7:124. doi: 10.1186/s40168-019-0739-x (PMC6716902; doi:10.1186/s40168-019-0739-x)
Supplement: Supplementary file 8 — Taxonomic classifications and top BLASTn matches (sources in parenthesis, sponge species names in italics) of common microbial OTUs between locations (Belize and Florida). (DOCX 38 kb) [file 40168_2019_739_MOESM8_ESM.docx]

**Additional file 8.** Taxonomic classifications and top BLASTn matches (sources in parenthesis, sponge specie names in italics) of common microbial OTUs between Locations (Belize and Florida). OTUs are ranked by percentage contributions up to 50% dissimilarity (SIMPER analysis) between collection locations and by OTU relative abundances in the sponge species. Only OTUs with significantly different (Metastats, P < 0.05) OTU abundances between locations are shown for each sponge species. Within the average relative abundance columns, --- indicates no sequences for that site and 0.00 indicates sequences made up less than 0.05 % of average sequence reads for that site. (DOCX)

|  | OTU | Top BLASTn Match (source) | Accession Number | % Identity | Contribution to Dissimilarity (%) | Metastats *P*-value | Ave. Relative Abd. (%) | |
| --- | --- | --- | --- | --- | --- | --- | --- | --- |
| Host |  |  |  |  |  |  | Florida | Belize |
| ***A. tubulata*** |  |  |  |  |  |  |  |  |
|  | 090 | Uncultured *Crenarchaeota* (*Agelas conifera*) | EU273912 | 100 | 1.22 | 0.0506 | 2.3 | 1.1 |
|  | 059 | Uncultured *Chloroflexi* (*Agelas dilatata*) | EF076128 | 100 | 1.20 | 0.0357 | 0.8 | 1.6 |
|  | 031 | Uncultured *Chloroflexi* (*Aplysina cauliformis*) | KF286150 | 100 | 1.19 | 0.0505 | 0.8 | 1.3 |
|  | 115 | Uncultured *Acidobacterium* sp. (*Agelas dilatata*) | EF076124 | 98 | 0.97 | 0.0072 | 1.6 | 0.6 |
|  | 118 | Uncultured *Nitrosococcus* (*Rhopaloeides odorabile*) | JN210811 | 98 | 0.78 | 0.0043 | 1.7 | 0.3 |
|  | 033 | Uncultured *Chloroflexi* (*Aplysina cauliformis*) | KF286135 | 99 | 0.73 | 0.0014 | --- | 0.4 |
|  | 002 | Uncultured *Ectothiorhodospiraceae* (*Amphimedon queenslandica*) | KX772780 | 99 | 0.54 | 0.0040 | 0.1 | 0.3 |
| ***V. gigantea*** |  |  |  |  |  |  |  |  |
|  | 064 | Uncultured *Acidobacterium* (*Chondrosia reniformis*) | KC200543 | 99 | 0.96 | 0.0549 | 1.1 | 1.5 |
|  | 060 | Uncultured *Gamma proteobacterium* (*Xestospongia muta*) | JN596632 | 100 | 0.94 | 0.0226 | 1.2 | 0.5 |
|  | 025 | Uncultured *Deferribacteres* (*Rhopaloeides odorabile*) | JN210882 | 100 | 0.87 | 0.0110 | 1.1 | 2.6 |
|  | 137 | Uncultured *Gamma proteobacterium* (*Neofibularia nolitangere*) | EU816812 | 99 | 0.65 | 0.0190 | 0.5 | 0.4 |
| ***V. reiswigi*** |  |  |  |  |  |  |  |  |
|  | 022 | Uncultured *Actinobacterium* (coral) | KC514272 | 100 | 1.60 | 0.0393 | 4.0 | 1.8 |
|  | 064 | Uncultured *Acidobacterium* (*Chondrosia reniformis*) | KC200543 | 99 | 0.94 | 0.0149 | 1.6 | 0.6 |
|  | 070 | Uncultured *Acidobacterium* (*Xestospongia testudinaria*) | JN596703 | 100 | 0.88 | 0.0044 | 1.8 | 0.5 |
|  | 167 | Uncultured *Gamma proteobacterium* (*Rhopaloeides odorabile*) | JN210809 | 100 | 0.78 | 0.0428 | 0.8 | 0.4 |
| ***X. muta*** |  |  |  |  |  |  |  |  |
|  | 016 | Uncultured *Deferribacteres* (*Xestospongia testudinaria*) | JN596760 | 100 | 1.34 | 0.0040 | 1.9 | 2.9 |
|  | 018 | Uncultured *Poribacteria* (*Aplysina cauliformis*) | KF286195 | 100 | 1.30 | 0.0051 | 1.4 | 3.9 |
|  | 035 | Uncultured *Nitrospira* (*Theonella* spp.) | MF039993 | 100 | 1.22 | 0.0179 | 2.2 | 1.6 |
|  | 036 | Uncultured *Actinobacterium* (coral) | KC514248 | 100 | 1.29 | 0.0228 | 1.8 | 2.8 |
|  | 037 | Uncultured *Delta proteobacterium* (*Xestospongia testudinaria*) | HQ270236 | 100 | 1.28 | 0.0311 | 1.8 | 2.3 |
|  | 050 | Uncultured *Actinobacterium* (coral) | KT715008 | 100 | 1.05 | 0.0062 | 2.1 | 1.1 |
|  | 065 | Uncultured *Bacteroidetes* (*Xestospongia muta*) | JN596623 | 100 | 1.15 | 0.0354 | 2.2 | 1.5 |
|  | 067 | Uncultured *Deferribacteres* (*Rhopaloeides odorabile*) | JN210871 | 100 | 0.91 | 0.0600 | 1.0 | 1.1 |
|  | 080 | Uncultured *Desulfovibrionales* (coral) | JQ516297 | 100 | 0.78 | 0.0393 | 1.0 | 0.7 |
|  | 081 | Uncultured *Chloroflexus* sp. (*Xestospongia muta*) | FJ481270 | 100 | 1.27 | 0.0033 | 1.5 | 2.9 |
|  | 103 | Uncultured *Gamma proteobacteria* (*Xestospongia muta*) | HQ270282 | 100 | 0.76 | 0.0490 | 1.2 | 0.6 |
|  | 125 | Uncultured *Gamma proteobacteria* (*Xestospongia muta*) | JN596624 | 100 | 0.54 | 0.0016 | 0.5 | 0.3 |
|  | 129 | Uncultured *Defluviicoccus* (*Theonella* spp.) | MF039926 | 99 | 0.52 | 0.0524 | 0.4 | 0.3 |
|  | 134 | Uncultured *Chloroflexi* (*Aplysina cauliformis*) | KF286207 | 99 | 0.60 | 0.0137 | 0.6 | 0.4 |
|  | 166 | Uncultured *Gemmatimondetes* (*Xestospongia muta*) | JN596602 | 100 | 0.68 | 0.0227 | 0.4 | 0.5 |
| ***C. plicifera*** |  |  |  |  |  |  |  |  |
|  | 006 | Uncultured *Synechococcus* (Seawater) | KX581283 | 100 | 2.59 | 0.0101 | 8.1 | 4.8 |
|  | 012 | Uncultured *Pelagibacterales* (Seawater) | KX844874 | 100 | 1.89 | 0.0000 | 2.7 | 4.6 |
|  | 024 | Uncultured *Flavobacteriales* (Seawater) | KT731809 | 100 | 1.63 | 0.0038 | 2.2 | 3.3 |
|  | 021 | Uncultured *Pelagibacterales* (Seawater) | KX844863 | 100 | 1.11 | 0.0036 | 1.0 | 1.7 |
|  | 023 | Uncultured *Flavobacteriaceae* (Seawater) | KM277349 | 100 | 1.10 | 0.0376 | 1.2 | 5.0 |
|  | 057 | Candidatus *Pelagibacter* (Seawater) | CP020778 | 100 | 1.08 | 0.0081 | 1.0 | 1.3 |
|  | 093 | *Gamma proteobacterium* (Seawater) | HQ675201 | 100 | 0.86 | 0.0486 | 0.7 | 9.8 |
|  | 073 | *Alpha proteobacterium* (Seawater) | HQ675181 | 100 | 0.86 | 0.0028 | 0.6 | 1.2 |
|  | 045 | Uncultured *Euryarchaeote* (Seawater) | KM277198 | 100 | 0.84 | 0.0009 | 1.1 | 0.3 |
|  | 107 | Uncultured *Rhodobacteraceae* (Seawater) | KF786682 | 100 | 0.75 | 0.0002 | 0.4 | 0.8 |
|  | 116 | *Alpha proteobacterium* (Seawater) | HQ675256 | 100 | 0.73 | 0.0000 | 0.4 | 0.7 |
|  | 113 | Uncultured *Rhodospirillaceae* (Oil Sheen) | KF786576 | 100 | 0.54 | 0.0009 | 1.2 | 0.8 |
|  | 155 | Uncultured *Gamma proteobacterium* (Oil Sheen) | KF786481 | 99 | 0.53 | 0.0004 | 0.2 | 0.4 |
|  | 162 | Uncultured *Bacteroidetes* (Seawater) | AY664244 | 100 | 0.53 | 0.0000 | 0.2 | 0.6 |
|  | 001 | Uncultured bacterium (*Niphates digitalis*) | JQ062789 | 100 | 0.48 | 0.0006 | 0.3 | 0.1 |
| ***C. vaginalis*** |  |  |  |  |  |  |  |  |
|  | 006 | Uncultured *Synechococcus* (Seawater) | KX581283 | 100 | 1.96 | 0.0076 | 3.1 | 1.0 |
|  | 066 | Uncultured *Gamma proteobacterium* (*Amphimedon queenslandica*) | KX772782 | 100 | 1.47 | 0.0071 | 4.1 | 0.3 |
|  | 015 | Uncultured *Gamma proteobacterium* (*Agelas dilatata*) | EF076162 | 100 | 1.39 | 0.0378 | 0.7 | 0.6 |
|  | 045 | Uncultured *Euryarchaeote* (Seawater) | KM277198 | 100 | 1.15 | 0.0416 | 0.9 | 0.4 |
| ***M. laxissima*** |  |  |  |  |  |  |  |  |
|  | 003 | Uncultured *Alpha proteobacterium*, *Plakortis* sp. (Seawater) | EF076075 | 98 | 17.62 | 0.0470 | 39.6 | 55.2 |
|  | 006 | Uncultured Synechococcus (Seawater) | KX581283 | 100 | 2.92 | 0.0100 | 3.8 | 0.5 |
|  | 079 | Uncultured *Planctomycete* (*Niphates* sp.) | JF443805 | 100 | 2.22 | 0.0031 | 2.1 | 0.3 |
|  | 194 | Uncultured *Actinomycetales* (*Haliclona simulans*) | FJ999615 | 100 | 1.16 | 0.0237 | 0.5 | 0.1 |
|  | 001 | Uncultured bacterium (*Niphates digitalis*) | JQ062789 | 100 | 1.11 | 0.0079 | 0.2 | 0.1 |
|  | 002 | Uncultured *Ectothiorhodospiraceae* (*Amphimedon queenslandica*) | KX772780 | 99 | 1.03 | 0.0036 | 0.2 | 0.1 |
|  | 058 | Uncultured *Rhodospirillaceae* (coral) | JQ516442 | 99 | 0.85 | 0.0576 | 5.0 | 0.0 |
|  | 198 | Uncultured *Synechococcus* sp. (Seawater) | KX581285 | 100 | 0.79 | 0.0092 | 0.3 | 0.0 |
|  | 160 | Uncultured *Rhodospirillaceae* (Oil sheen) | KF786399 | 100 | 0.63 | 0.0004 | 0.0 | 0.1 |
|  | 197 | Uncultured *Rhodospirillaceae* (Seawater) | JQ515043 | 100 | 0.63 | 0.0112 | 0.1 | 0.1 |
| ***N. digitalis*** |  |  |  |  |  |  |  |  |
|  | 006 | Uncultured *Synechococcus* (Seawater) | KX581283 | 100 | 2.47 | 0.0111 | 4.7 | 1.1 |
|  | 011 | Uncultured *Alpha proteobacterium* (Seawater) | KM580275 | 100 | 1.45 | 0.0275 | 1.3 | 0.5 |
|  | 034 | Uncultured *SAR406* (Oil sheen) | KF786970 | 100 | 1.12 | 0.0268 | 0.6 | 0.3 |
|  | 044 | Uncultured *SAR86* (Seawater) | EU361687 | 100 | 1.04 | 0.0527 | 0.5 | 0.3 |
|  | 028 | Uncultured *Gamma proteobacterium* (Oil sheen) | KF786735 | 100 | 0.92 | 0.0320 | 0.5 | 0.2 |
|  | 003 | Uncultured *Alpha proteobacterium*, *Plakortis* sp. (Seawater) | EF076075 | 98 | 0.91 | 0.0527 | 0.3 | 0.2 |
|  | 045 | Uncultured *Euryarchaeote* (Seawater) | KM277198 | 100 | 0.85 | 0.0114 | 0.6 | 0.1 |
|  | 043 | Uncultured *Rhodospirillaceae* (Oil sheen) | KF786444 | 98 | 0.80 | 0.0000 | 0.1 | 0.3 |
| **Seawater** |  |  |  |  |  |  |  |  |
|  | 026 | *Prochlorococcus* sp. (Seawater) | CP018346 | 100 | 2.30 | 0.0007 | 2.7 | 7.0 |
|  | 006 | Uncultured *Synechococcus* (Seawater) | KX581283 | 100 | 2.27 | 0.0005 | 7.2 | 3.3 |
|  | 011 | Uncultured *Alpha proteobacterium* (Seawater) | KM580275 | 100 | 2.21 | 0.0366 | 4.8 | 3.8 |
|  | 028 | Uncultured *Gamma proteobacterium* (Oil sheen) | KF786735 | 100 | 1.86 | 0.0218 | 3.2 | 2.7 |
|  | 029 | Uncultured *Gamma proteobacterium* (Seawater) | LC018959 | 100 | 1.70 | 0.0093 | 2.8 | 2.2 |
|  | 012 | Uncultured *Pelagibacterales* (Seawater) | KX844874 | 100 | 1.47 | 0.0189 | 1.8 | 3.8 |
|  | 045 | Uncultured *Euryarchaeote* (Seawater) | KM277198 | 100 | 1.40 | 0.0169 | 2.7 | 1.4 |
|  | 003 | Uncultured *Alpha proteobacterium*, *Plakortis* sp. (Seawater) | EF076075 | 98 | 1.31 | 0.0094 | 2.7 | 1.1 |
|  | 073 | *Alpha proteobacterium* (Seawater) | HQ675181 | 100 | 0.98 | 0.0023 | 0.6 | 1.0 |
|  | 044 | Uncultured *SAR86 gamma proteobacterium* (Seawater) | EU361687 | 100 | 0.86 | 0.0130 | 0.8 | 0.6 |
|  | 128 | *Pseudomalteromonas* sp. (Seawater) | MG799456 | 100 | 0.83 | 0.0227 | 0.4 | 2.5 |
|  | 156 | Uncultured *SAR324* cluster bacterium (Seawater) | KX987574 | 100 | 0.74 | 0.0003 | 0.3 | 0.7 |
|  | 197 | Uncultured *Rhodospirillaceae* (Seawater) | JQ515043 | 100 | 0.61 | 0.0011 | 0.4 | 0.3 |
|  | 133 | Uncultured *Gamma proteobacterium* (Seawater) | KX581298 | 100 | 0.61 | 0.0205 | 0.5 | 0.3 |
|  | 184 | *Vibrio neocaledonicus* (Seawater) | MG905403 | 100 | 0.52 | 0.0398 | 0.1 | 0.9 |
|  | 201 | Uncultured *Gamma proteobacterium* (Seawater) | JF949216 | 100 | 0.51 | 0.0190 | 0.2 | 0.2 |
|  | 111 | Uncultured *Alpha proteobacterium* (Seawater) | KJ870971 | 99 | 0.51 | 0.0027 | 0.6 | 0.2 |
|  | 286 | *Alpha proteobacterium* (Seawater) | HQ675229 | 100 | 0.50 | 0.0522 | 0.2 | 0.3 |
|  | 116 | *Alpha proteobacterium* (Seawater) | HQ675256 | 100 | 0.50 | 0.0461 | --- | 0.0 |
