# Supplementary material for: Testing the relationship between microbiome composition and flux of carbon and nutrients in Caribbean coral reef sponges
Source: Microbiome. 2019 Aug 29;7:124. doi: 10.1186/s40168-019-0739-x (PMC6716902; doi:10.1186/s40168-019-0739-x)
Supplement: Supplementary file 9 — Spearman (rank order) correlations between carbon (DOC, POC) and nutrient flux (NH4, NOx, and PO4 specific filtration rates, μmol/s/L sponge) and microbial community (alpha) diversity metrics, showing correlation coefficients (R) and significance (P) values. (DOCX 20 kb) [file 40168_2019_739_MOESM9_ESM.docx]

**Additional file 9.** Spearman (rank order) correlations between carbon (DOC, POC) and nutrient flux (NH_4_, NO_x_, and PO_4_ specific filtration rates, μmol/s/L sponge) and microbial community (alpha) diversity metrics, showing correlation coefficients (*R*) and significance (*P*) values. (DOCX)

|  | **OTU Richness (S)** | | **Shannon-Weaver (H’)** | | **Simpson (D)** | |
| --- | --- | --- | --- | --- | --- | --- |
| **Correlate** | *R* | *P* | *R* | *P* | *R* | *P* |
| DOC | 0.093 | 0.409 | 0.189 | 0.093 | -0.201 | 0.075 |
| POC | 0.409 | <0.001* | 0.002 | 0.983 | 0.091 | 0.411 |
| NH_4_ | 0.040 | 0.833 | 0.552 | <0.001* | -0.609 | <0.001* |
| NO_x_ | -0.094 | 0.620 | -0.253 | 0.176 | 0.233 | 0.212 |
| PO_4_ | -0.298 | 0.109 | -0.147 | 0.436 | 0.105 | 0.579 |
