# Supplementary material for: Testing the relationship between microbiome composition and flux of carbon and nutrients in Caribbean coral reef sponges
Source: Microbiome. 2019 Aug 29;7:124. doi: 10.1186/s40168-019-0739-x (PMC6716902; doi:10.1186/s40168-019-0739-x)
Supplement: Supplementary file 10 — Highly correlated microbial abundances and NH4 specific filtration rates for all sponges with more than 2 individuals sampled in Belize with a significant P-value (< 0.05). (DOCX 33 kb) [file 40168_2019_739_MOESM10_ESM.docx]

**Additional file 10.** Highly correlated microbial abundances and NH_4_ specific filtration rates for all sponges with more than 2 individuals sampled in Belize with a significant *P*-value (< 0.05). Taxonomy is based on the SILVA database. Within the average relative abundance columns, --- indicates no sequences for that site and 0.00 indicates sequences made up less than 0.01 % of average sequence reads for that site. (DOCX)

| Host | OTU | Phylum | Lowest Taxonomic Identification | Pearson Correlation | P-value | Ave. Relative Abd. (%)  Florida Belize | |
| --- | --- | --- | --- | --- | --- | --- | --- |
| ***A. tubulata*** | 001 | *Proteobacteria* | *G. Roseivivax* | -0.9528 | 0.0472 | 0.17 | 0.10 |
|  | 006 | *Cyanobacteria* | *G. Synechococcus* | -0.9761 | 0.0239 | 0.32 | 0.07 |
|  | 012 | *Proteobacteria* | *F. Pelagibacteraceae* | -0.9590 | 0.0410 | 0.02 | 0.11 |
|  | 030 | *Proteobacteria* | *F. Pelagibacteraceae* | -0.9862 | 0.0138 | 0.05 | 0.04 |
|  | 043 | *Proteobacteria* | *F. Rhodobacteraceae* | -0.9457 | 0.0543 | 0.05 | 0.03 |
|  | 082 | *Proteobacteria* | *F. Syntrophobacteraceae* | 0.9589 | 0.0411 | 1.77 | 1.47 |
|  | 087 | *Proteobacteria* | *F. Rhodobacteraceae* | -0.9584 | 0.0416 | 0.02 | 0.02 |
|  | 090 | *Crenarchaeota* | *G. Cenarcheum* | 0.9974 | 0.0026 | 2.30 | 1.11 |
|  | 114 | *Gemmatimonadetes* | *C. Gemm-2* | -0.9732 | 0.0268 | 0.04 | 0.03 |
|  | 144 | *Euryarchaeota* | *C. Thermoplasmata* | -0.9420 | 0.0580 | 0.00 | 0.01 |
|  | 160 | *Proteobacteria* | *C. Alpha proteobacteria* | -0.9832 | 0.0168 | 0.01 | 0.01 |
|  | 238 | *Proteobacteria* | *F. Rhodospirillaceae* | -0.9420 | 0.0580 | 0.01 | 0.04 |
|  | 249 | *Acidobacteria* | *O. Solibacterales* | 0.9786 | 0.0214 | 0.04 | 0.02 |
|  | 470 | *Proteobacteria* | *G. Bdellovibrio* | 0.9786 | 0.0214 | 0.04 | 0.01 |
|  | 483 | *PAUC34F* | *P. PAUC34F* | 0.9786 | 0.0214 | 0.01 | 0.01 |
|  | 561 | *Proteobacteria* | *G. Thiohalorhabdales* | -0.9535 | 0.0465 | 0.02 | 0.01 |
|  | 568 | *Planctomycetes* | *F. Pirellulaceae* | -0.9420 | 0.0580 | 0.04 | 0.00 |
|  | 609 | *Chloroflexi* | *C. Anaerolineae* | -0.9786 | 0.0214 | --- | 0.01 |
|  | 656 | *Proteobacteria* | *C. Alpha proteobacteria* | -0.9786 | 0.0214 | 0.00 | 0.01 |
|  | 852 | *Proteobacteria* | *C. Gamma proteobacteria* | -0.9802 | 0.0198 | --- | 0.01 |
|  |  |  |  |  |  |  |  |
| ***X. muta*** | 011 | *Proteobacteria* | *C. Alpha proteobacteria* | -0.8864 | 0.0452 | 0.03 | 0.04 |
|  | 021 | *Proteobacteria* | *G. Pelagibacteraceae* | -0.9410 | 0.0171 | 0.01 | 0.02 |
|  | 043 | *Proteobacteria* | *F. Rhodobacteraceae* | -0.9228 | 0.0255 | 0.01 | 0.05 |
|  | 146 | *Chloroflexi* | *---* | 0.8858 | 0.0455 | 0.11 | 0.06 |
|  | 178 | *Gemmatimonadetes* | *C. Gemm-4* | 0.9290 | 0.0225 | 0.14 | 0.04 |
|  | 194 | *Actinobacteria* | *O. Actinomycetales* | 0.9117 | 0.0311 | 0.02 | 0.01 |
|  | 208 | *Unknown* | *K. Bacteria* | -0.9552 | 0.0113 | 0.05 | 0.06 |
|  | 264 | *Unknown* | *K. Bacteria* | 0.9161 | 0.0288 | 0.01 | 0.00 |
|  | 271 | *Planctomycetes* | *F. Phycisphaeraceae* | 0.9161 | 0.0288 | 0.01 | 0.00 |
|  | 298 | *Proteobacteria* | *C. Alpha proteobacteria* | 0.9161 | 0.0288 | 0.01 | 0.00 |
|  | 311 | *Proteobacteria* | *C. Beta proteobacteria* | 0.9550 | 0.0114 | 0.01 | 0.01 |
|  | 327 | *Bacteroidetes* | *P. Bacteroidetes* | 0.9449 | 0.0154 | 0.02 | 0.05 |
|  | 339 | *Proteobacteria* | *C. Alpha proteobacteria* | 0.9161 | 0.0288 | 0.01 | 0.00 |
|  | 341 | *SAR406* | *C. AB16* | 0.9161 | 0.0288 | --- | 0.00 |
|  | 343 | *Chloroflexi* | *C. SAR202* | 0.9350 | 0.0197 | 0.05 | 0.03 |
|  | 370 | *Proteobacteria* | *F. Pelagibacteraceae* | 0.9161 | 0.0288 | 0.03 | 0.00 |
|  | 372 | *Gemmatimonadetes* | *C. Gemm-2* | -0.9161 | 0.0288 | --- | 0.01 |
|  | 400 | *Proteobacteria* | *F. Piscirickettsiaceae* | 0.9080 | 0.0330 | 0.01 | 0.01 |
|  | 419 | *Proteobacteria* | *P. Proteobacteria* | 0.9161 | 0.0288 | 0.00 | 0.00 |
|  | 434 | *Proteobacteria* | *F. Cystobacterineae* | 0.9999 | <0.0001 | 0.00 | 0.01 |
|  | 538 | *Proteobacteria* | *F. Pelagibacteraceae* | 0.9161 | 0.0288 | 0.00 | 0.00 |
|  | 571 | *Planctomycetes* | *F. Pirellulaceae* | 0.9161 | 0.0288 | --- | 0.00 |
|  | 581 | *Proteobacteria* | *F. Rhodospirillaceae* | 0.9161 | 0.0288 | 0.00 | 0.00 |
|  | 602 | *Proteobacteria* | *C. Gamma proteobacteria* | 0.9235 | 0.0251 | 0.01 | 0.03 |
|  | 608 | *Proteobacteria* | *C. Gamma proteobacteria* | 0.9161 | 0.0288 | --- | 0.00 |
|  | 609 | *Chloroflexi* | *C. Anaerolineae* | 0.9146 | 0.0296 | --- | 0.03 |
|  | 625 | *Proteobacteria* | *C. Gamma proteobacteria* | 0.9161 | 0.0288 | 0.00 | 0.00 |
|  | 647 | *Chloroflexi* | *C. SAR202* | 0.9161 | 0.0288 | 0.01 | 0.01 |
|  | 670 | *Verrucomicrobia* | *G. Rubritalea* | 0.9161 | 0.0288 | --- | 0.00 |
|  | 715 | *Proteobacteria* | *C. Delta proteobacteria* | 0.9161 | 0.0288 | 0.00 | 0.00 |
|  | 819 | *Proteobacteria* | *F. Rhodospirillaceae* | 0.9161 | 0.0288 | --- | 0.00 |
|  | 831 | *Proteobacteria* | *P. Proteobacteria* | 0.9161 | 0.0288 | --- | 0.00 |
|  | 855 | *Proteobacteria* | *C. Alpha proteobacteria* | 0.9161 | 0.0288 | --- | 0.00 |
|  | 904 | *Chloroflexi* | *C. TK17* | 0.9161 | 0.0288 | 0.00 | 0.00 |
|  | 907 | *Chloroflexi* | *C. Anaerolineae* | 0.9161 | 0.0288 | --- | 0.00 |
|  | 922 | *Proteobacteria* | *P. Proteobacteria* | 0.9161 | 0.0288 | --- | 0.00 |
|  | 928 | *Euryarchaeota* | *C. Thermoplasmata* | 0.9161 | 0.0288 | --- | 0.00 |
|  | 941 | *Proteobacteria* | *G. Paracoccus* | 0.9161 | 0.0288 | --- | 0.00 |
|  | 943 | *SAR406* | *C. AB16* | 0.9161 | 0.0288 | --- | 0.00 |
|  | 956 | *Proteobacteria* | *C. Delta proteobacteria* | 0.9161 | 0.0288 | --- | 0.00 |
|  | 992 | *Cyanobacteria* | *G. Synechococcus* | 0.8921 | 0.0419 | 0.00 | 0.01 |
|  | 1000 | *Proteobacteria* | *C. Gamma proteobacteria* | 0.9080 | 0.0330 | 0.02 | 0.01 |
|  |  |  |  |  |  |  |  |
| ***C. plicifera*** | 071 | *Crenarchaeota* | *G. Nitrosopumilus* | 0.9273 | 0.0233 | 0.02 | 0.01 |
|  | 087 | *Proteobacteria* | *F. Rhodobacteraceae* | 0.9128 | 0.0305 | 0.75 | 0.60 |
|  | 119 | *Proteobacteria* | *C. Gamma proteobacteria* | -0.9576 | 0.0104 | 0.02 | 0.01 |
|  | 120 | *Proteobacteria* | *G. Candidatus Portiera* | 0.8844 | 0.0463 | 0.42 | 0.46 |
|  | 137 | *Proteobacteria* | *F. Ectothiorhodospiraceae* | 0.9576 | 0.0104 | 0.01 | 0.01 |
|  | 145 | *Proteobacteria* | *F. Endozoicomonaceae* | -0.9474 | 0.0143 | 0.27 | 0.22 |
|  | 165 | *Proteobacteria* | *G. Rhodovulum* | -0.9020 | 0.0363 | 0.03 | 0.02 |
|  | 173 | *PAUC34f* | *P. PAUC34f* | 0.9020 | 0.0363 | 0.00 | 0.01 |
|  | 195 | *Proteobacteria* | *C. Alpha proteobacteria* | -0.8672 | 0.0569 | 0.00 | 0.03 |
|  | 262 | *Proteobacteria* | *C. Gamma proteobacteria* | 0.9003 | 0.0372 | 0.01 | 0.01 |
|  | 372 | *Proteobacteria* | *C. Alpha proteobacteria* | 0.9169 | 0.0284 | 0.02 | 0.12 |
|  | 387 | *Proteobacteria* | *G. Bdellovibrio* | -0.9026 | 0.0360 | 0.03 | 0.01 |
|  | 397 | *Proteobacteria* | *F. Pelagibacteraceae* | 0.9454 | 0.0152 | 0.05 | --- |
|  | 448 | *Proteobacteria* | *F. Pelagibacteraceae* | -0.8681 | 0.0563 | 0.01 | 0.02 |
|  | 505 | *Chloroflexi* | *C. SAR202* | 0.9235 | 0.0251 | 0.00 | 0.01 |
|  | 536 | *Proteobacteria* | *O. Myxococcales* | 0.9235 | 0.0251 | 0.01 | 0.01 |
|  | 543 | *Proteobacteria* | *F. Pseudomonadaceae* | 0.9576 | 0.0104 | --- | 0.01 |
|  | 601 | *Cyanobacteria* | *G. Synechococcus* | 0.9003 | 0.0372 | 0.01 | 0.01 |
|  | 611 | *Proteobacteria* | *F. Rickettsiales* | 0.9026 | 0.0360 | 0.01 | 0.01 |
|  | 650 | *Parvarchaeota* | *C. Parvarchaea* | -0.9003 | 0.0372 | 0.01 | 0.01 |
|  | 653 | *Proteobacteria* | *F. Pelagibacteraceae* | 0.9288 | 0.0226 | 0.01 | 0.01 |
|  | 788 | *Proteobacteria* | *O. Rhizobiales* | 0.9258 | 0.0240 | 0.00 | 0.01 |
|  | 955 | *Proteobacteria* | *F. Rhodospirillaceae* | -0.9003 | 0.0372 | 0.01 | 0.01 |
|  |  |  |  |  |  |  |  |
| ***C. vaginalis*** | 013 | *Proteobacteria* | *C. Alpha proteobacteria* | -0.8844 | 0.0464 | 0.03 | 0.02 |
|  | 046 | *Proteobacteria* | *F. Rhodobacteraceae* | 0.9536 | 0.0119 | 0.07 | 0.12 |
|  | 052 | *Proteobacteria* | *C. Gamma proteobacteria* | -0.9010 | 0.0369 | 0.08 | 0.04 |
|  | 054 | *Proteobacteria* | *C. Poribacteria* | -0.9325 | 0.0208 | 0.01 | 0.00 |
|  | 065 | *Bacteroidetes* | *G. Salisaeta* | -0.8799 | 0.0491 | 0.01 | 0.01 |
|  | 071 | *Crenarchaeota* | *G. Nitrosopumilus* | 0.8816 | 0.0480 | 0.02 | 0.00 |
|  | 131 | *Chloroflexi* | *C. SAR202* | 0.8816 | 0.0480 | 0.01 | 0.00 |
|  | 152 | *Chloroflexi* | *C. TK17* | -0.9325 | 0.0208 | 0.00 | 0.00 |
|  | 173 | *PAUC34f* | *P. PAUC34f* | 0.8859 | 0.0455 | 0.01 | 0.01 |
|  | 325 | *Bacteroidetes* | *P. Bacteroidetes* | -0.8816 | 0.0480 | 0.02 | 0.02 |
|  | 371 | *Proteobacteria* | *F. Pelagibacteraceae* | -0.9549 | 0.0114 | 0.02 | 0.03 |
|  | 381 | *Proteobacteria* | *F. Rhodobacteraceae* | 0.9581 | 0.0102 | 0.01 | 0.01 |
|  | 403 | *Proteobacteria* | *F. Pelagibacteraceae* | -0.9581 | 0.0102 | 0.02 | 0.02 |
|  | 430 | *Proteobacteria* | *F. Pelagibacteraceae* | -0.9532 | 0.0121 | 0.00 | 0.01 |
|  | 452 | *Proteobacteria* | *F. Pelagibacteraceae* | -0.9312 | 0.0214 | --- | 0.01 |
|  | 494 | *Unknown* | *K. Bacteria* | -0.8859 | 0.0455 | 0.02 | 0.07 |
|  | 605 | *Proteobacteria* | *C. Alpha proteobacteria* | -0.8816 | 0.0480 | --- | 0.00 |
|  | 606 | *Proteobacteria* | *C. Gamma proteobacteria* | -0.9741 | 0.0050 | 0.00 | 0.01 |
|  | 683 | *Proteobacteria* | *F. Pelagibacteraceae* | -0.9522 | 0.0125 | 0.01 | 0.01 |
|  | 836 | *Proteobacteria* | *F. Coxiellaceae* | 0.8816 | 0.0480 | --- | 0.00 |
|  | 906 | *Proteobacteria* | *F. Pelagibacteraceae* | 0.8816 | 0.0480 | --- | 0.00 |
|  |  |  |  |  |  |  |  |
| ***M. laxissima*** | 014 | *Proteobacteria* | *C. Alpha proteobacteria* | -0.9995 | 0.0204 | 0.03 | 0.02 |
|  | 015 | *Proteobacteria* | *F. Ectothiorhodospiraceae* | -0.9998 | 0.0130 | 0.03 | 0.03 |
|  | 226 | *Bacteroidetes* | *G. Roseivirga* | 0.9995 | 0.0204 | 0.08 | 0.70 |
|  |  |  |  |  |  |  |  |
| ***N. digitalis*** | 04 | *Proteobacteria* | *F. Pelagibacteraceae* | 0.8978 | 0.0386 | 4.60 | 2.69 |
|  | 05 | *Proteobacteria* | *F. Endozoicomonaceae* | 0.8845 | 0.0463 | 0.15 | 0.08 |
|  | 08 | *Actinobacteria* | *O. Acidimicrobiales* | 0.9260 | 0.0239 | 1.29 | 1.01 |
|  | 011 | *Proteobacteria* | *C. Alpha proteobacteria* | 0.9379 | 0.0184 | 1.30 | 0.54 |
|  | 013 | *Proteobacteria* | *C. Alpha proteobacteria* | 0.9417 | 0.0168 | 0.03 | 0.02 |
|  | 021 | *Proteobacteria* | *F. Pelagibacteraceae* | 0.9749 | 0.0047 | 0.33 | 0.24 |
|  | 028 | *Proteobacteria* | *G. Candidatus Portiera* | 0.8830 | 0.0472 | 0.47 | 0.21 |
|  | 045 | *Euryarchaeota* | *C. Thermoplasmata* | 0.9041 | 0.0351 | 0.57 | 0.14 |
|  | 056 | *Cyanobacteria* | *G. Synechococcus* | -0.9622 | 0.0088 | 0.02 | 0.01 |
|  | 063 | *Chloroflexi* | *C. SAR202* | 0.9622 | 0.0088 | 0.01 | 0.00 |
|  | 075 | *Bacteroidetes* | *F. Cryomorphaceae* | 0.9641 | 0.0081 | 0.30 | 0.12 |
|  | 087 | *Proteobacteria* | *F. Rhodobacteraceae* | 0.8881 | 0.0441 | 0.24 | 0.25 |
|  | 116 | *Proteobacteria* | *F. Pelagibacteraceae* | 0.8917 | 0.0421 | 0.15 | 0.16 |
|  | 133 | *Proteobacteria* | *O. Alteromonadales* | 0.9135 | 0.0302 | 0.07 | 0.04 |
|  | 199 | *Proteobacteria* | *G. Halomonas* | 0.9476 | 0.0143 | --- | 0.01 |
|  | 206 | *Proteobacteria* | *F. Pelagibacteraceae* | 0.9276 | 0.0231 | 0.04 | 0.03 |
|  | 245 | *Bacteroidetes* | *F. Cryomorphaceae* | 0.9256 | 0.0241 | 0.05 | 0.03 |
|  | 286 | *Proteobacteria* | *F. Rickettsiales* | 0.9227 | 0.0255 | 0.01 | 0.02 |
|  | 291 | *Proteobacteria* | *F. Rhodospirillaceae* | 0.9980 | 0.0001 | 0.01 | 0.01 |
|  | 312 | *Proteobacteria* | *F. Pelagibacteraceae* | 0.9980 | 0.0001 | 0.01 | 0.01 |
|  | 363 | *Proteobacteria* | *F. Pelagibacteraceae* | 0.9136 | 0.0301 | 0.01 | 0.02 |
|  | 368 | *Proteobacteria* | *F. Pelagibacteraceae* | 0.9955 | 0.0004 | 0.08 | 0.08 |
|  | 374 | *Bacteroidetes* | *F. Flavobacteriaceae* | 0.8910 | 0.0425 | 0.00 | 0.01 |
|  | 424 | *Proteobacteria* | *F. Rhodospirillaceae* | 0.9476 | 0.0143 | 0.01 | 0.01 |
|  | 471 | *Proteobacteria* | *F. Pelagibacteraceae* | 0.9090 | 0.0325 | 0.01 | 0.01 |
|  | 524 | *Proteobacteria* | *F. Pelagibacteraceae* | 0.9622 | 0.0088 | 0.01 | 0.00 |
|  | 548 | *Planctomycetes* | *F. Phycisphaeraceae* | 0.8895 | 0.0434 | --- | 0.01 |
|  | 552 | *Bacteroidetes* | *F. Flavobacteriaceae* | 0.9622 | 0.0088 | 0.01 | 0.00 |
|  | 593 | *Proteobacteria* | *G. Bdellovibrio* | 0.9865 | 0.0019 | 0.00 | 0.01 |
|  | 610 | *Unknown* | *K. Bacteria* | 0.9622 | 0.0088 | --- | 0.01 |
|  | 639 | *Bacteroidetes* | *F. Flavobacteriaceae* | 0.9622 | 0.0088 | 0.00 | 0.00 |
|  | 821 | *Proteobacteria* | *F. Rhodospirillaceae* | 0.9622 | 0.0088 | --- | 0.00 |
|  | 836 | *Proteobacteria* | *F. Coxiellaceae* | 0.9622 | 0.0088 | --- | 0.00 |
|  |  |  |  |  |  |  |  |

* *P* phylum, *C* class, *O* order, *F* family, *G* genus
