# Supplementary material for: Testing the relationship between microbiome composition and flux of carbon and nutrients in Caribbean coral reef sponges
Source: Microbiome. 2019 Aug 29;7:124. doi: 10.1186/s40168-019-0739-x (PMC6716902; doi:10.1186/s40168-019-0739-x)
Supplement: Supplementary file 11 — Significant differences in correlated microbial abundance and POC specific filtration rates for all sponges sampled at both sites that had more than 2 individuals sampled at each site and a significant P-value (< 0.05). (DOCX 56 kb) [file 40168_2019_739_MOESM11_ESM.docx]

**Additional file 11.** Significant differences in correlated microbial abundance and POC specific filtration rates for all sponges sampled at both sites that had more than 2 individuals sampled at each site and a significant *P*-value (< 0.05). Taxonomy is based on the SILVA database. Within the average relative abundance columns, --- indicates no sequences for that site and 0.00 indicates sequences made up less than 0.01 % of average sequence reads for that site. (DOCX)

| Host | OTU | Phylum | Lowest Taxonomic Identification | Pearson Correlation | P-value | Ave. Relative Abd. (%)  Florida Belize | |
| --- | --- | --- | --- | --- | --- | --- | --- |
| ***A. tubulata*** | 084 | *Proteobacteria* | *G. Candidatus Portiera* | 0.6601 | 0.0378 | 0.03 | 0.02 |
|  | 129 | *Proteobacteria* | *C. Alpha proteobacteria* | 0.8645 | 0.0012 | 0.17 | 0.04 |
|  | 137 | *Chloroflexi* | *F. Caldilineaceae* | 0.6592 | 0.0381 | 0.06 | 0.05 |
|  | 141 | *Chloroflexi* | *C. SAR202* | 0.7223 | 0.0183 | 0.03 | 0.02 |
|  | 142 | *Chloroflexi* | *F. Caldilineaceae* | 0.6548 | 0.0399 | 0.83 | 0.56 |
|  | 173 | *PAUC34f* | *P. PAUC34f* | 0.7670 | 0.0096 | 0.01 | 0.01 |
|  | 181 | *Proteobacteria* | *O. Spirobacillales* | 0.8857 | 0.0007 | 0.62 | 0.01 |
|  | 185 | *Poribacteria* | *P. Poribacteria* | 0.7189 | 0.0191 | 0.04 | 0.02 |
|  | 236 | *Proteobacteria* | *G. Haliangium* | 0.8591 | 0.0015 | 0.01 | --- |
|  | 258 | *Verrucomicrobia* | *C. Pedosphaerae* | 0.8971 | 0.0004 | 0.42 | --- |
|  | 261 | *Actinobacteria* | *O. Acidimicrobiales* | 0.7624 | 0.0104 | 0.01 | --- |
|  | 293 | *Proteobacteria* | *G. Bdellovibrio* | 0.8689 | 0.0011 | 0.36 | 0.01 |
|  | 294 | *Proteobacteria* | *C. Gamma proteobacteria* | 0.6629 | 0.0367 | 0.03 | 0.01 |
|  | 298 | *Proteobacteria* | *C. Alpha proteobacteria* | 0.8270 | 0.0032 | 0.01 | --- |
|  | 299 | *Acidobacteria* | *P. Acidobacteria* | 0.6522 | 0.0410 | 0.03 | 0.00 |
|  | 304 | *Proteobacteria* | *C. Gamma proteobacteria* | 0.6485 | 0.0425 | 0.02 | 0.00 |
|  | 317 | *Firmicutes* | *G. Lactobacillus* | 0.8560 | 0.0016 | 0.15 | 0.10 |
|  | 323 | *Chloroflexi* | *C. SAR202* | 0.8450 | 0.0021 | 0.01 | --- |
|  | 330 | *Proteobacteria* | *O. Spirobacillales* | 0.9335 | <0.0001 | 0.00 | --- |
|  | 348 | *Unknown* | *K. Bacteria* | 0.6842 | 0.0291 | 0.01 | 0.00 |
|  | 353 | *Actinobacteria* | *O. Acidimicrobiales* | 0.7320 | 0.0161 | 0.01 | --- |
|  | 358 | *Chloroflexi* | *C. SAR202* | 0.7748 | 0.0085 | 0.02 | 0.01 |
|  | 360 | *Proteobacteria* | *C. Gamma proteobacteria* | 0.7237 | 0.0180 | 0.14 | 0.05 |
|  | 375 | *Proteobacteria* | *F. Pelagibacteraceae* | 0.8722 | 0.0010 | 0.00 | 0.00 |
|  | 396 | *Actinobacteria* | *O. Acidimicrobiales* | 0.7577 | 0.0111 | 0.01 | 0.01 |
|  | 427 | *Proteobacteria* | *O. Alteromonadales* | 0.8722 | 0.0010 | 0.00 | --- |
|  | 444 | *Proteobacteria* | *G. Bdellovibrio* | 0.7523 | 0.0121 | 0.11 | 0.00 |
|  | 456 | *Proteobacteria* | *G. Bdellovibrio* | 0.6559 | 0.0395 | 0.06 | 0.01 |
|  | 461 | *PAUC34f* | *P. PAUC34f* | -0.7074 | 0.0221 | 0.00 | 0.01 |
|  | 473 | *Proteobacteria* | *F. Pelagibacteraceae* | 0.7389 | 0.0146 | 0.00 | --- |
|  | 512 | *Unknown* | *K. Archaea* | 0.8722 | 0.0010 | 0.00 | --- |
|  | 537 | *Chloroflexi* | *C. SAR202* | 0.8305 | 0.0029 | 0.00 | --- |
|  | 570 | *Parvarchaeota* | *C. Parvarchaea* | 0.8086 | 0.0046 | 0.02 | 0.02 |
|  | 575 | *Chloroflexi* | *C. Anaerolineae* | 0.8178 | 0.0038 | 0.00 | --- |
|  | 585 | *SAR406* | *C. AB16* | 0.8722 | 0.0010 | 0.00 | 0.00 |
|  | 592 | *Proteobacteria* | *F. Piscirickettsiaceae* | 0.6611 | 0.0374 | 0.00 | 0.00 |
|  | 621 | *Unknown* | *K. Bacteria* | 0.7989 | 0.0050 | 0.01 | --- |
|  | 650 | *Actinobacteria* | *O. Acidimicrobiales* | 0.8722 | 0.0010 | 0.00 | 0.00 |
|  | 652 | *Proteobacteria* | *G. Plesiocystis* | 0.7389 | 0.0146 | 0.00 | --- |
|  | 658 | *Proteobacteria* | *F. Pelagibacteraceae* | 0.8722 | 0.0010 | 0.00 | --- |
|  | 663 | *Planctomycetes* | *F. Phycisphaeraceae* | 0.8722 | 0.0010 | 0.00 | --- |
|  | 677 | *Proteobacteria* | *G. Bdellovibrio* | 0.8722 | 0.0010 | 0.00 | --- |
|  | 700 | *Proteobacteria* | *C. Gamma proteobacteria* | 0.7024 | 0.0235 | 0.02 | 0.00 |
|  | 733 | *Proteobacteria* | *F. Piscirickettsiaceae* | 0.7389 | 0.0146 | 0.00 | 0.00 |
|  | 742 | *Unknown* | *K. Bacteria* | 0.7389 | 0.0146 | 0.00 | --- |
|  | 796 | *Acidobacteria* | *P. Acidobacteria* | 0.7875 | 0.0068 | 0.02 | 0.01 |
|  | 807 | *Planctomycetes* | *F. Pirellulaceae* | 0.8722 | 0.0010 | 0.00 | --- |
|  | 825 | *Proteobacteria* | *P. Proteobacteria* | 0.7500 | 0.0125 | 0.00 | --- |
|  | 835 | *Bacteroidetes* | *F. Flammeovirgaceae* | 0.8722 | 0.0010 | 0.00 | --- |
|  | 878 | *Bacteroidetes* | *F. Flavobacteriaceae* | 0.8722 | 0.0010 | 0.00 | --- |
|  | 882 | *Chloroflexi* | *C. Anaerolineae* | 0.7389 | 0.0146 | 0.00 | --- |
|  | 886 | *Chloroflexi* | *C. TK17* | 0.7094 | 0.0216 | 0.01 | 0.00 |
|  | 973 | *Proteobacteria* | *C. Alpha proteobacteria* | 0.8722 | 0.0010 | 0.00 | --- |
|  | 997 | *Proteobacteria* | *F. Alteromonadaceae* | 0.8722 | 0.0010 | 0.00 | --- |
|  | 1000 | *Proteobacteria* | *C. Gamma proteobacteria* | 0.8722 | 0.0010 | 0.00 | 0.00 |
|  |  |  |  |  |  |  |  |
| ***V. gigantea*** | 005 | *Proteobacteria* | *F. Endozoicomonaceae* | 0.7835 | 0.0214 | 0.11 | 0.10 |
|  | 006 | *Cyanobacteria* | *G. Synechococcus* | 0.8745 | 0.0045 | 0.11 | 0.02 |
|  | 020 | *Cyanobacteria* | *G. Synechococcus* | 0.8051 | 0.0159 | 3.55 | 0.18 |
|  | 030 | *Proteobacteria* | *F. Pelagibacteraceae* | 0.8897 | 0.0031 | 0.01 | --- |
|  | 039 | *Chloroflexi* | *C. TK17* | -0.8073 | 0.0154 | 0.09 | 0.17 |
|  | 046 | *Proteobacteria* | *F. Rhodobacteraceae* | -0.7095 | 0.0487 | 0.02 | 0.04 |
|  | 052 | *Proteobacteria* | *C. Gamma proteobacteria* | 0.8555 | 0.0068 | 3.90 | 3.44 |
|  | 060 | *Proteobacteria* | *F. Piscirickettsiaceae* | 0.7149 | 0.0463 | 1.24 | 0.51 |
|  | 071 | *Crenarchaeota* | *G. Nitrosopumilus* | 0.7934 | 0.0188 | 0.13 | --- |
|  | 075 | *Bacteroidetes* | *F. Cryomorphaceae* | 0.7427 | 0.0348 | 0.01 | 0.00 |
|  | 089 | *Chloroflexi* | *C. SAR202* | -0.7367 | 0.0371 | 1.61 | 1.89 |
|  | 092 | *Proteobacteria* | *F. Rhodospirillaceae* | 0.8496 | 0.0076 | 0.47 | 0.32 |
|  | 129 | *Proteobacteria* | *F. Rhodospirillaceae* | -0.7414 | 0.0353 | 0.38 | 0.43 |
|  | 145 | *Proteobacteria* | *F. Endozoicomonaceae* | 0.8700 | 0.0050 | 0.07 | 0.01 |
|  | 147 | *Proteobacteria* | *F. Rhodospirillaceae* | 0.8384 | 0.0093 | 0.43 | 0.16 |
|  | 154 | *Actinobacteria* | *P. Actinobacteria* | 0.7599 | 0.0287 | 0.40 | 0.33 |
|  | 160 | *Proteobacteria* | *C. Alpha proteobacteria* | -0.7742 | 0.0241 | 0.01 | 0.01 |
|  | 193 | *Proteobacteria* | *F. Endozoicomonaceae* | 0.7955 | 0.0182 | 0.00 | --- |
|  | 213 | *Proteobacteria* | *C. Alpha proteobacteria* | 0.8286 | 0.0110 | 0.55 | 0.03 |
|  | 214 | *Bacteroidetes* | *O. Flavobacteriales* | -0.7582 | 0.0292 | 0.01 | 0.01 |
|  | 251 | *Proteobacteria* | *O. Chromatiales* | 0.8452 | 0.0082 | 0.09 | --- |
|  | 256 | *Proteobacteria* | *F. Rhodobacteraceae* | 0.7183 | 0.0447 | 0.01 | 0.00 |
|  | 257 | *Proteobacteria* | *F. Pelagibacteraceae* | 0.8052 | 0.0159 | 0.01 | --- |
|  | 274 | *Proteobacteria* | *F. Spirobacillales* | 0.7155 | 0.0460 | 0.01 | 0.00 |
|  | 357 | *Proteobacteria* | *G. Bdellovibrio* | 0.8554 | 0.0068 | 0.01 | --- |
|  | 394 | *Actinobacteria* | *P. Actinobacteria* | -0.7841 | 0.0213 | 0.01 | 0.02 |
|  | 417 | *Proteobacteria* | *C. Alpha proteobacteria* | -0.7133 | 0.0470 | 0.04 | 0.05 |
|  | 429 | *Verrucomicrobia* | *F. Verrucomicrobiaceae* | 0.9507 | 0.0003 | 0.01 | 0.00 |
|  | 487 | *Verrucomicrobia* | *G. Persicirhabdus* | 0.8679 | 0.0052 | 0.00 | --- |
|  | 574 | *Bacteroidetes* | *F. Flammeovirgaceae* | 0.7664 | 0.0265 | 0.04 | 0.01 |
|  | 601 | *Cyanobacteria* | *G. Synechococcus* | 0.8679 | 0.0052 | 0.00 | --- |
|  | 626 | *Proteobacteria* | *G. Candidatus Portiera* | 0.7980 | 0.0176 | 0.00 | --- |
|  | 652 | *Proteobacteria* | *G. Plesiocystis* | 0.8679 | 0.0052 | 0.00 | --- |
|  | 655 | *Proteobacteria* | *F. Piscirickettsiaceae* | 0.8405 | 0.0090 | 0.00 | --- |
|  | 693 | *Lentisphaerae* | *P. Lentisphaerae* | 0.8679 | 0.0052 | 0.00 | --- |
|  | 759 | *Proteobacteria* | *P. Proteobacteria* | 0.8679 | 0.0052 | 0.00 | --- |
|  | 904 | *Chloroflexi* | *C. TK17* | 0.7424 | 0.0349 | 0.00 | --- |
|  | 922 | *Proteobacteria* | *P. Proteobacteria* | 0.8679 | 0.0052 | 0.00 | --- |
|  | 926 | *Chloroflexi* | *C. Anaerolineae* | 0.8679 | 0.0052 | 0.01 | --- |
|  | 955 | *Proteobacteria* | *F. Rhodospirillaceae* | 0.8679 | 0.0052 | 0.00 | --- |
|  | 967 | *Proteobacteria* | *C. Gamma proteobacteria* | 0.7113 | 0.0479 | 0.01 | 0.00 |
|  | 979 | *Spirochaetes* | *G. Turneriella* | 0.7980 | 0.0176 | 0.00 | --- |
|  |  |  |  |  |  |  |  |
| ***V. reiswigi*** | 013 | *Proteobacteria* | *C. Alpha proteobacteria* | -0.8758 | 0.0222 | 0.04 | 0.01 |
|  | 028 | *Proteobacteria* | *G. Candidatus Portiera* | 0.8331 | 0.0394 | 0.01 | 0.03 |
|  | 031 | *Chloroflexi* | *C. SAR202* | 0.8798 | 0.0208 | 1.72 | 2.51 |
|  | 061 | *Proteobacteria* | *C. Alpha proteobacteria* | 0.8901 | 0.0175 | 0.01 | 0.02 |
|  | 069 | *Acidobacteria* | *P. Acidobacteria* | -0.8638 | 0.0266 | 1.61 | 0.16 |
|  | 070 | *Acidobacteria* | *P. Acidobacteria* | -0.8370 | 0.0377 | 1.78 | 0.45 |
|  | 087 | *Proteobacteria* | *F. Rhodobacteraceae* | -0.8141 | 0.0486 | 0.02 | 0.01 |
|  | 099 | *Acidobacteria* | *F. PAUC26f* | -0.8935 | 0.0164 | 0.09 | 0.05 |
|  | 117 | *Proteobacteria* | *C. Gamma proteobacteria* | -0.8453 | 0.0340 | 0.59 | 0.26 |
|  | 134 | *Acidobacteria* | *P. Acidobacteria* | -0.8700 | 0.0242 | 0.86 | 0.24 |
|  | 146 | *Chloroflexi* | *C. TK17* | -0.8280 | 0.0418 | 0.15 | 0.03 |
|  | 149 | *Chloroflexi* | *O. TK18* | -0.8494 | 0.0323 | 0.47 | 0.09 |
|  | 154 | *Actinobacteria* | *P. Actinobacteria* | -0.8164 | 0.0475 | 0.34 | 0.02 |
|  | 163 | *Chloroflexi* | *O. TK18* | -0.8166 | 0.0474 | 0.05 | --- |
|  | 178 | *Gemmatimonadetes* | *C. Gemm-4* | -0.8182 | 0.0466 | 0.46 | 0.01 |
|  | 204 | *Chloroflexi* | *C. SAR202* | 0.8273 | 0.0422 | 0.03 | 0.07 |
|  | 211 | *Acidobacteria* | *P. Acidobacteria* | -0.8286 | 0.0416 | 0.37 | 0.11 |
|  | 236 | *Proteobacteria* | *G. Haliangium* | -0.8403 | 0.0362 | 0.01 | --- |
|  | 251 | *Proteobacteria* | *O. Chromatiales* | -0.8403 | 0.0362 | 0.02 | --- |
|  | 252 | *Proteobacteria* | *C. Gamma proteobacteria* | -0.8475 | 0.0331 | 0.40 | 0.14 |
|  | 300 | *Chloroflexi* | *C. Ktedonobacteria* | -0.8327 | 0.0397 | 0.08 | 0.02 |
|  | 323 | *Chloroflexi* | *C. SAR202* | -0.8239 | 0.0438 | 0.04 | 0.00 |
|  | 329 | *Proteobacteria* | *C. Alpha proteobacteria* | -0.8131 | 0.0491 | 0.01 | --- |
|  | 379 | *Thermi* | *G. B-42* | -0.8348 | 0.0387 | 0.08 | --- |
|  | 383 | *Acidobacteria* | *P. Acidobacteria* | -0.8131 | 0.0491 | 0.01 | --- |
|  | 392 | *Gemmatimonadetes* | *C. Gemm-2* | -0.8403 | 0.0362 | 0.01 | --- |
|  | 401 | *Chloroflexi* | *C. TK17* | -0.8403 | 0.0362 | 0.01 | --- |
|  | 415 | *Proteobacteria* | *C. Gamma proteobacteria* | -0.8403 | 0.0362 | 0.01 | --- |
|  | 473 | *Bacteroidetes* | *O. Flavobacteriales* | -0.8403 | 0.0362 | 0.01 | --- |
|  | 505 | *Chloroflexi* | *C. SAR202* | 0.8490 | 0.0325 | --- | 0.00 |
|  | 520 | *Planctomycetes* | *C. Pla3* | 0.8791 | 0.0211 | --- | 0.01 |
|  | 615 | *Verrucomicrobia* | *G. Pelagicoccus* | -0.8454 | 0.0340 | 0.03 | --- |
|  | 624 | *Proteobacteria* | *C. Delta proteobacteria* | -0.8403 | 0.0362 | 0.01 | --- |
|  | 730 | *Proteobacteria* | *O. Spirobacillales* | -0.8403 | 0.0362 | 0.01 | --- |
|  | 775 | *Chloroflexi* | *C. SAR202* | 0.8131 | 0.0491 | 0.00 | 0.01 |
|  | 816 | *Proteobacteria* | *C. Gamma proteobacteria* | -0.8383 | 0.0371 | 0.02 | --- |
|  |  |  |  |  |  |  |  |
| ***X. muta*** | 007 | *Crenarchaeota* | *G. Nitrosopumilus* | 0.6870 | 0.0282 | 8.16 | 7.11 |
|  | 010 | *Chloroflexi* | *C. SAR202* | -0.7606 | 0.0106 | 2.32 | 3.30 |
|  | 031 | *Chloroflexi* | *C. SAR202* | -0.6700 | 0.0340 | 0.96 | 1.09 |
|  | 035 | *Nitrospirae* | *F. Nitrospiraceae* | 0.6880 | 0.0279 | 2.25 | 1.61 |
|  | 058 | *Proteobacteria* | *F. Rhodospirillaceae* | 0.6675 | 0.0349 | 0.08 | --- |
|  | 059 | *Chloroflexi* | *C. SAR202* | -0.6727 | 0.0330 | 0.58 | 0.72 |
|  | 066 | *Proteobacteria* | *G. Candidatus Portiera* | 0.7998 | 0.0055 | 0.02 | 0.00 |
|  | 067 | *AncK6* | *P. AncK6* | -0.6387 | 0.0469 | 0.96 | 1.13 |
|  | 078 | *Proteobacteria* | *C. Beta proteobacteria* | 0.7979 | 0.0057 | 0.01 | 0.00 |
|  | 081 | *Chloroflexi* | *O. TK18* | -0.6472 | 0.0431 | 1.54 | 2.89 |
|  | 129 | *Proteobacteria* | *F. Rhodospirillaceae* | 0.8114 | 0.0044 | 0.45 | 0.30 |
|  | 132 | *Acidobacteria* | *P. Acidobacteria* | 0.6894 | 0.0274 | 0.04 | --- |
|  | 134 | *Chloroflexi* | *O. TK17* | 0.6884 | 0.0277 | 0.04 | 0.39 |
|  | 167 | *Proteobacteria* | *O. Chromatiales* | 0.9454 | <0.0001 | 0.08 | 0.04 |
|  | 170 | *Proteobacteria* | *C. Alpha proteobacteria* | 0.8213 | 0.0036 | 0.01 | --- |
|  | 171 | *Chloroflexi* | *C. SAR202* | 0.7444 | 0.0135 | 0.23 | 0.09 |
|  | 181 | *Proteobacteria* | *O. Spirobacillales* | 0.7448 | 0.0134 | 0.16 | 0.05 |
|  | 191 | *Proteobacteria* | *G. Novosphingobium* | 0.9004 | 0.0004 | 0.01 | --- |
|  | 200 | *Actinobacteria* | *O. Acidomicrobiales* | 0.6884 | 0.0277 | 0.08 | 0.12 |
|  | 202 | *Unknown* | *K. Bacteria* | 0.8788 | 0.0008 | 0.09 | 0.02 |
|  | 213 | *Proteobacteria* | *C. Alpha proteobacteria* | 0.7040 | 0.0231 | 0.01 | 0.00 |
|  | 224 | *Proteobacteria* | *F. Rhodobacteraceae* | 0.7519 | 0.0121 | 0.03 | 0.01 |
|  | 230 | *Chloroflexi* | *C. Anaerolineae* | 0.6689 | 0.0344 | 0.02 | 0.02 |
|  | 238 | *Proteobacteria* | *F. Rhodospirillaceae* | 0.8345 | 0.0027 | 0.11 | 0.04 |
|  | 249 | *Acidobacteria* | *F. PAUC26f* | 0.6510 | 0.0415 | 0.10 | 0.04 |
|  | 251 | *Proteobacteria* | *O. Chromatiales* | 0.6538 | 0.0403 | 0.03 | 0.00 |
|  | 253 | *Proteobacteria* | *G. Bdellovibrio* | 0.6602 | 0.0377 | 0.02 | 0.01 |
|  | 256 | *Proteobacteria* | *F. Rhodobacteraceae* | 0.7743 | 0.0086 | 0.33 | 0.14 |
|  | 278 | *Bacteroidetes* | *F. Flammeovirgaceae* | 0.8252 | 0.0033 | 0.00 | --- |
|  | 284 | *Bacteroidetes* | *O. Flavobacteriales* | 0.7571 | 0.0112 | 0.01 | --- |
|  | 295 | *Chloroflexi* | *C. SAR202* | 0.6655 | 0.0357 | 0.04 | 0.01 |
|  | 313 | *Proteobacteria* | *C. Gamma proteobacteria* | 0.7353 | 0.0154 | 0.01 | 0.01 |
|  | 323 | *Chloroflexi* | *C. SAR202* | 0.8560 | 0.0016 | 0.29 | 0.02 |
|  | 326 | *Proteobacteria* | *C. Alpha proteobacteria* | 0.7719 | 0.0089 | 0.04 | 0.01 |
|  | 345 | *Crenarchaeota* | *F. Cenarchaeaceae* | 0.8252 | 0.0033 | 0.00 | --- |
|  | 346 | *Unknown* | *H. Bacteria* | 0.8922 | 0.0005 | 0.01 | 0.00 |
|  | 360 | *Proteobacteria* | *C. Gamma proteobacteria* | 0.7700 | 0.0092 | 0.03 | 0.00 |
|  | 380 | *Proteobacteria* | *F. Piscirickettsiaceae* | 0.7115 | 0.0210 | 0.01 | 0.00 |
|  | 401 | *Acidobacteria* | *P. Acidobacteria* | 0.9489 | <0.0001 | 0.00 | --- |
|  | 426 | *PAUC34f* | *P. PAUC34f* | 0.7254 | 0.0176 | 0.08 | 0.07 |
|  | 444 | *Proteobacteria* | *G. Bdellovibrio* | 0.7031 | 0.0233 | 0.01 | --- |
|  | 446 | *Chloroflexi* | *C. Anaerolineae* | 0.7887 | 0.0067 | 0.01 | --- |
|  | 456 | *Proteobacteria* | *G. Bdellovibrio* | 0.6738 | 0.0327 | 0.01 | --- |
|  | 462 | *Chloroflexi* | *O. TK18* | 0.9656 | <0.0001 | 0.01 | 0.00 |
|  | 493 | *Proteobacteria* | *F. Rhodospirillaceae* | 0.6385 | 0.0469 | 0.09 | 0.04 |
|  | 508 | *Bacteroidetes* | *F. Rhodothermaceae* | 0.8003 | 0.0054 | 0.01 | --- |
|  | 514 | *Proteobacteria* | *F. Pelagibacteraceae* | 0.8252 | 0.0033 | 0.00 | --- |
|  | 522 | *Planctomycetes* | *C. Pla3* | 0.9489 | <0.0001 | 0.01 | --- |
|  | 526 | *Proteobacteria* | *G. Bdellovibrio* | 0.6962 | 0.0253 | 0.01 | --- |
|  | 529 | *Proteobacteria* | *G. Bdellovibrio* | 0.9655 | <0.0001 | 0.02 | --- |
|  | 545 | *Proteobacteria* | *G. Bdellovibrio* | 0.7229 | 0.0182 | 0.01 | 0.00 |
|  | 554 | *Crenarchaeota* | *G. Nitrosopumilus* | 0.8252 | 0.0033 | 0.00 | --- |
|  | 574 | *Proteobacteria* | *O. Rhizobiales* | 0.9489 | <0.0001 | 0.00 | --- |
|  | 648 | *Parvarchaeota* | *C. Parvarchaea* | 0.7396 | 0.0145 | 0.03 | 0.01 |
|  | 663 | *Planctomycetes* | *O. Phycisphaerales* | 0.8252 | 0.0033 | 0.00 | --- |
|  | 672 | *Proteobacteria* | *O. Rhodospirillales* | 0.8252 | 0.0033 | 0.00 | --- |
|  | 674 | *Chloroflexi* | *C. SAR202* | 0.8474 | 0.0020 | 0.01 | --- |
|  | 744 | *Chloroflexi* | *C. Anaerolineae* | 0.6489 | 0.0424 | 0.02 | 0.01 |
|  | 794 | *Acidobacteria* | *P. Acidobacteria* | 0.8627 | 0.0013 | 0.01 | --- |
|  | 801 | *Proteobacteria* | *G. Bdellovibrio* | 0.8303 | 0.0029 | 0.03 | --- |
|  | 854 | *Chloroflexi* | *C. SAR202* | -0.6522 | 0.0410 | 0.01 | 0.01 |
|  | 902 | *Chlamydiae* | *O. Chlamydiales* | 0.8252 | 0.0033 | 0.00 | --- |
|  | 937 | *Chloroflexi* | *C. SAR202* | 0.8252 | 0.0033 | 0.00 | --- |
|  | 981 | *Proteobacteria* | *F. Piscirickettsiaceae* | 0.8252 | 0.0033 | 0.00 | --- |
|  | 989 | *Chloroflexi* | *C. SAR202* | 0.8252 | 0.0033 | 0.00 | --- |
|  | 996 | *Proteobacteria* | *G. Vibrio* | 0.8252 | 0.0033 | 0.00 | --- |
|  | 1000 | *Proteobacteria* | *C. Gamma proteobacteria* | 0.8634 | 0.0013 | 0.02 | 0.01 |
|  |  |  |  |  |  |  |  |
| ***C. plicifera*** | 047 | *Proteobacteria* | *F. Entotheonellaceae* | -0.6771 | 0.0156 | 0.02 | 0.01 |
|  | 078 | *Proteobacteria* | *C. Beta proteobacteria* | 0.5892 | 0.0438 | 3.14 | 0.12 |
|  | 083 | *Proteobacteria* | *C. Gamma proteobacteria* | 0.5858 | 0.0453 | 0.03 | 0.02 |
|  | 164 | *Chloroflexi* | *C. SAR202* | 0.7155 | 0.0089 | 0.01 | 0.00 |
|  | 222 | *Actinobacteria* | *P. Actinobacteria* | -0.6339 | 0.0269 | 0.11 | 0.22 |
|  | 269 | *SAR406* | *C. AB16* | -0.6229 | 0.0305 | 0.08 | 0.14 |
|  | 278 | *Bacteroidetes* | *F. Flammeovirgaceae* | 0.5870 | 0.0448 | 0.01 | 0.00 |
|  | 334 | *Unknown* | *K. Bacteria* | 0.6796 | 0.0150 | 0.10 | 0.17 |
|  | 360 | *Proteobacteria* | *C. Gamma proteobacteria* | 0.6647 | 0.0184 | 0.00 | 0.00 |
|  | 433 | *Proteobacteria* | *F. Piscirickettsiaceae* | 0.5815 | 0.0473 | 0.05 | 0.03 |
|  | 466 | *Proteobacteria* | *F. Bacteriovoracaceae* | -0.6664 | 0.0180 | 0.01 | 0.02 |
|  | 473 | *Bacteroidetes* | *O. Flavobacteriales* | 0.5806 | 0.0477 | 0.03 | 0.02 |
|  | 483 | *PAUC34f* | *P. PAUC34f* | 0.6292 | 0.0284 | 0.00 | 0.00 |
|  | 510 | *Bacteroidetes* | *F. Saprospiraeceae* | 0.6223 | 0.0307 | 0.02 | 0.01 |
|  | 581 | *Proteobacteria* | *O. Alteromonadales* | 0.7415 | 0.0058 | 0.01 | 0.01 |
|  | 730 | *Proteobacteria* | *O. Spirobacillales* | 0.6667 | 0.0179 | 0.01 | 0.00 |
|  | 784 | *Proteobacteria* | *C. Delta proteobacteria* | -0.6260 | 0.0295 | 0.00 | 0.01 |
|  | 821 | *Proteobacteria* | *F. Rhodospirillaceae* | -0.6043 | 0.0374 | --- | 0.01 |
|  | 944 | *Bacteroidetes* | *G. Fluviicola* | 0.6216 | 0.0310 | 0.00 | 0.00 |
|  |  |  |  |  |  |  |  |
| ***C. vaginalis*** | 058 | *Proteobacteria* | *F. Rhodospirillaceae* | 0.7109 | 0.0212 | 0.01 | --- |
|  | 065 | *Bacteroidetes* | *G. Salisaeta* | 0.9574 | <0.0001 | 0.01 | 0.01 |
|  | 142 | *Chloroflexi* | *F. Caldilineaceae* | 0.8089 | 0.0046 | 0.01 | 0.00 |
|  | 158 | *Proteobacteria* | *F. SAR324* | 0.8491 | 0.0019 | 0.01 | 0.00 |
|  | 177 | *Chloroflexi* | *C. SAR202* | 0.8404 | 0.0023 | 0.01 | --- |
|  | 202 | *Actinobacteria* | *O. Acidomicrobiales* | 0.7885 | 0.0067 | 0.05 | 0.01 |
|  | 220 | *Actinobacteria* | *P. Actinobacteria* | 0.8827 | 0.0007 | 0.01 | 0.00 |
|  | 243 | *Proteobacteria* | *F. Rhodobacteraceae* | 0.7746 | 0.0085 | 0.00 | 0.00 |
|  | 290 | *Verrucomicrobia* | *O. Puniceicoccales* | 0.6970 | 0.0251 | 0.01 | 0.01 |
|  | 325 | *Chloroflexi* | *C. SAR202* | 0.8710 | 0.0010 | 0.02 | 0.02 |
|  | 337 | *Unknown* | *K. Bacteria* | 0.7431 | 0.0138 | 0.01 | --- |
|  | 343 | *Chloroflexi* | *C. SAR202* | 0.8367 | 0.0025 | 0.00 | 0.00 |
|  | 364 | *Proteobacteria* | *F. Rhodospirillaceae* | 0.8367 | 0.0025 | 0.00 | 0.00 |
|  | 365 | *Bacteroidetes* | *P. Bacteroidetes* | 0.06491 | 0.0423 | 0.01 | 0.00 |
|  | 406 | *Chloroflexi* | *C. Ktedonobacteria* | 0.9567 | <0.0001 | 0.00 | --- |
|  | 411 | *Chloroflexi* | *C. Anaerolineae* | 0.9567 | <0.0001 | 0.00 | --- |
|  | 415 | *Proteobacteria* | *C. Gamma proteobacteria* | 0.8329 | 0.0028 | 0.00 | --- |
|  | 422 | *Unknown* | *K. Bacteria* | 0.7354 | 0.0154 | 0.01 | --- |
|  | 494 | *Unknown* | *K. Bacteria* | 0.8968 | 0.0004 | 0.01 | 0.01 |
|  | 511 | *Chloroflexi* | *C. S085* | 0.9567 | <0.0001 | 0.00 | --- |
|  | 527 | *Proteobacteria* | *F. Bacteriovoracaceae* | 0.6441 | 0.0444 | 0.01 | 0.00 |
|  | 553 | *Crenarchaeota* | *F. Ectothiorhodospiraceae* | 0.7074 | 0.0221 | 0.01 | --- |
|  | 564 | *Proteobacteria* | *C. Gamma proteobacteria* | 0.9567 | <0.0001 | 0.00 | --- |
|  | 577 | *Verrucomicrobia* | *G. Coraliomargarita* | 0.7963 | 0.0058 | 0.01 | --- |
|  | 580 | *Proteobacteria* | *C. Alpha proteobacteria* | 0.9567 | <0.0001 | 0.00 | --- |
|  | 600 | *Proteobacteria* | *G. Bdellovibrio* | 0.9567 | <0.0001 | 0.00 | --- |
|  | 646 | *Proteobacteria* | *P. Proteobacteria* | 0.6449 | 0.0441 | 0.00 | 0.00 |
|  | 678 | *Proteobacteria* | *F. Pelagibacteraceae* | 0.9567 | <0.0001 | 0.00 | --- |
|  | 751 | *Bacteroidetes* | *P. Bacteroidetes* | 0.7069 | 0.0223 | 0.00 | 0.00 |
|  | 793 | *Proteobacteria* | *G. Bdellovibrio* | 0.9567 | <0.0001 | 0.00 | --- |
|  | 841 | *Proteobacteria* | *C. Alpha proteobacteria* | 0.9567 | <0.0001 | 0.00 | --- |
|  | 863 | *Proteobacteria* | *F. Piscirickettsiaceae* | 0.9567 | <0.0001 | 0.00 | --- |
|  | 884 | *Proteobacteria* | *G. Bdellovibrio* | 0.9567 | <0.0001 | 0.00 | --- |
|  | 891 | *Chlamydiae* | *F. Simkaniaceae* | 0.9567 | <0.0001 | 0.00 | --- |
|  | 899 | *Proteobacteria* | *P. Proteobacteria* | 0.9567 | <0.0001 | 0.00 | --- |
|  | 907 | *Chloroflexi* | *C. Anaerolineae* | 0.9567 | <0.0001 | 0.00 | --- |
|  | 947 | *Proteobacteria* | *F. Bacteriovoracaceae* | 0.9567 | <0.0001 | 0.00 | --- |
|  | 950 | *Verrucomicrobia* | *C. Verruco-5* | 0.6449 | 0.0441 | 0.00 | 0.00 |
|  | 959 | *Proteobacteria* | *O. Myxococcales* | 0.9567 | <0.0001 | 0.00 | --- |
|  |  |  |  |  |  |  |  |
| ***M. laxissima*** | 004 | *Proteobacteria* | *F. Pelagibacteraceae* | 0.6472 | 0.0314 | 0.20 | 0.05 |
|  | 023 | *Bacteroidetes* | *F. Cryomorphaceae* | -0.6363 | 0.0353 | 0.05 | 0.18 |
|  | 030 | *Proteobacteria* | *F. Pelagibacteraceae* | 0.7377 | 0.0096 | 0.05 | 0.03 |
|  | 035 | *Nitrospirae* | *F. Nitrospiraceae* | 0.6023 | 0.0499 | 0.02 | 0.01 |
|  | 049 | *Proteobacteria* | *C. Beta proteobacteria* | 0.7305 | 0.0107 | 0.02 | 0.01 |
|  | 081 | *Chloroflexi* | *O. TK18* | -0.7568 | 0.0070 | 0.01 | 0.01 |
|  | 086 | *Actinobacteria* | *O. Acidomicrobiales* | -0.8027 | 0.0029 | 0.00 | 0.01 |
|  | 088 | *Proteobacteria* | *F. Pelagibacteraceae* | -0.7783 | 0.0048 | --- | 0.07 |
|  | 118 | *Proteobacteria* | *F. Ectothiorhodospiraceae* | 0.6860 | 0.0198 | 0.01 | --- |
|  | 130 | *Bacteroidetes* | *F. Flammeovirgaceae* | -0.7560 | 0.0071 | 0.65 | 1.51 |
|  | 140 | *Unknown* | *K. Bacteria* | 0.6930 | 0.0181 | 0.01 | --- |
|  | 153 | *Proteobacteria* | *F. Pelagibacteraceae* | -0.7266 | 0.0113 | --- | 0.02 |
|  | 179 | *Chloroflexi* | *C. SAR202* | 0.6330 | 0.0366 | 0.89 | --- |
|  | 181 | *Proteobacteria* | *O. Spirobacillales* | 0.6051 | 0.0486 | 0.01 | --- |
|  | 243 | *Unknown* | *K. Bacteria* | -0.6531 | 0.0293 | --- | 0.00 |
|  | 257 | *Proteobacteria* | *F. Pelagibacteraceae* | -0.7412 | 0.0091 | --- | 0.01 |
|  | 287 | *Proteobacteria* | *C. Gamma proteobacteria* | -0.7266 | 0.0113 | --- | 0.00 |
|  | 307 | *Proteobacteria* | *O. Rhodobacterales* | -0.6415 | 0.0334 | 0.01 | 0.01 |
|  | 309 | *Proteobacteria* | *F. Enterobacteriaceae* | 0.6160 | 0.0436 | 0.00 | --- |
|  | 353 | *Actinobacteria* | *O. Acidomicrobiales* | 0.6318 | 0.0370 | 0.00 | --- |
|  | 356 | *Proteobacteria* | *O. Rhizobiales* | 0.6128 | 0.0450 | 0.01 | 0.00 |
|  | 362 | *Proteobacteria* | *F. Rhodospirillaceae* | -0.7635 | 0.0063 | --- | 0.02 |
|  | 367 | *Proteobacteria* | *C. Delta proteobacteria* | -0.7266 | 0.0113 | --- | 0.00 |
|  | 388 | *Bacteroidetes* | *G. Formosa* | -0.6151 | 0.0440 | --- | 0.00 |
|  | 404 | *Proteobacteria* | *G. Pseudidiomarina* | -0.6753 | 0.0226 | --- | 0.01 |
|  | 418 | *Proteobacteria* | *C. Alpha proteobacteria* | -0.6447 | 0.0322 | 0.00 | 0.00 |
|  | 457 | *Proteobacteria* | *G. Pseudoalteromonas* | -0.8077 | 0.0026 | 0.01 | 0.11 |
|  | 491 | *Proteobacteria* | *F. Coxiellaceae* | -0.7608 | 0.0066 | --- | 0.06 |
|  | 518 | *Proteobacteria* | *F. Rhodospirillaceae* | -0.7124 | 0.0139 | 0.00 | 0.01 |
|  | 527 | *Proteobacteria* | *F. Bacteriovoracaceae* | -0.7266 | 0.0113 | --- | 0.00 |
|  | 538 | *Proteobacteria* | *F. Pelagibacteraceae* | -0.7266 | 0.0113 | --- | 0.01 |
|  | 539 | *Bacteroidetes* | *F. Cryomorphaceae* | -0.7266 | 0.0113 | --- | 0.00 |
|  | 548 | *Planctomycetes* | *F. Pirellulaceae* | -0.6665 | 0.0251 | --- | 0.02 |
|  | 571 | *Planctomycetes* | *F. Pirellulaceae* | -0.7822 | 0.0044 | 0.00 | 0.04 |
|  | 590 | *Proteobacteria* | *C. Alpha proteobacteria* | -0.7266 | 0.0113 | --- | 0.00 |
|  | 608 | *Unknown* | *K. Bacteria* | -0.7266 | 0.0113 | --- | 0.05 |
|  | 639 | *Proteobacteria* | *F. Coxiellaceae* | -0.7266 | 0.0113 | --- | 0.00 |
|  | 643 | *Proteobacteria* | *C. Beta proteobacteria* | -0.6806 | 0.0212 | --- | 0.00 |
|  | 680 | *Proteobacteria* | *F. Coxiellaceae* | -0.7266 | 0.0113 | --- | 0.00 |
|  | 716 | *Chlamydiae* | *O. Chlamydiales* | -0.7347 | 0.0100 | --- | 0.01 |
|  | 754 | *Proteobacteria* | *G. Candidatus Portiera* | -0.6246 | 0.0399 | 0.00 | 0.02 |
|  | 782 | *Actinobacteria* | *O. Acidomicrobiales* | -0.7266 | 0.0113 | --- | 0.00 |
|  | 802 | *Proteobacteria* | *G. Coxiella* | -0.7266 | 0.0113 | --- | 0.03 |
|  | 827 | *Proteobacteria* | *G. Nisaea* | -0.7458 | 0.0084 | --- | 0.02 |
|  | 831 | *Proteobacteria* | *P. Proteobacteria* | -0.7266 | 0.0113 | --- | 0.03 |
|  | 844 | *Proteobacteria* | *G. Delftia* | -0.6915 | 0.0184 | 0.00 | 0.01 |
|  | 877 | *Proteobacteria* | *C. Gamma proteobacteria* | -0.7266 | 0.0113 | --- | 0.00 |
|  | 883 | *Proteobacteria* | *C. Gamma proteobacteria* | -0.7019 | 0.0161 | --- | 0.01 |
|  | 890 | *Planctomycetes* | *C. OM190* | -0.7266 | 0.0113 | --- | 0.00 |
|  | 918 | *Chlamydiae* | *F. Simkaniaceae* | -0.6920 | 0.0183 | --- | 0.01 |
|  | 932 | *Proteobacteria* | *C. Gamma proteobacteria* | -0.7364 | 0.0098 | --- | 0.01 |
|  | 941 | *Proteobacteria* | *F. Pelagibacteraceae* | -0.7266 | 0.0113 | --- | 0.00 |
|  | 958 | *Unknown* | *K. Bacteria* | -0.7857 | 0.0041 | --- | 0.01 |
|  | 960 | *Verrucomicrobia* | *G. Rubritalea* | -0.7266 | 0.0113 | --- | 0.00 |
|  |  |  |  |  |  |  |  |
| ***N. digitalis*** | 091 | *Chloroflexi* | *C. SAR202* | 0.1665 | 0.0328 | 0.01 | 0.00 |
|  | 100 | *Proteobacteria* | *C. Gamma proteobacteria* | 0.5899 | 0.0435 | 0.01 | 0.01 |
|  | 361 | *Proteobacteria* | *G. Marinobacter* | 0.6381 | 0.0256 | --- | 0.00 |
|  | 409 | *Proteobacteria* | *C. Gamma proteobacteria* | 0.5889 | 0.0439 | 0.00 | --- |
|  | 451 | *Proteobacteria* | *G. Oleibacter* | 0.6381 | 0.0256 | --- | 0.00 |
|  | 458 | *Proteobacteria* | *G. Oleispira* | 0.6381 | 0.0256 | --- | 0.00 |
|  | 498 | *Proteobacteria* | *F. Piscirickettsiaceae* | 0.6129 | 0.0341 | 0.00 | 0.00 |
|  | 506 | *Proteobacteria* | *P. Proteobacteria* | 0.6443 | 0.0237 | 0.01 | 0.01 |
|  | 560 | *Bacteroidetes* | *F. Cryomorphaceae* | 0.6381 | 0.0256 | --- | 0.00 |
|  | 646 | *Proteobacteria* | *P. Proteobacteria* | 0.6199 | 0.0315 | --- | 0.03 |
|  | 719 | *Proteobacteria* | *G. Bacteriovorax* | 0.6381 | 0.0256 | --- | 0.00 |
|  | 722 | *Proteobacteria* | *F. Pelagibacteraceae* | 0.5929 | 0.0422 | 0.00 | --- |
|  | 757 | *Proteobacteria* | *P. Proteobacteria* | 0.7026 | 0.0108 | 0.00 | 0.00 |
|  | 815 | *Chloroflexi* | *C. SAR202* | 0.6381 | 0.0256 | --- | 0.00 |
|  | 874 | *Proteobacteria* | *G. Bdellovibrio* | 0.6381 | 0.0256 | --- | 0.00 |
|  | 892 | *Proteobacteria* | *C. Alpha proteobacteria* | 0.6381 | 0.0256 | --- | 0.00 |
|  | 899 | *Proteobacteria* | *P. Proteobacteria* | 0.6381 | 0.0256 | --- | 0.00 |

* *P* phylum, *C* class, *O* order, *F* family, *G* genus
